# Transcriptional networks in plasmacytoid dendritic cells stimulated with synthetic TLR 7 agonists

**DOI:** 10.1186/1471-2172-8-26

**Published:** 2007-10-12

**Authors:** Woubalem Birmachu, Raymond M Gleason, Barbara J Bulbulian, Christie L Riter, John P Vasilakos, Kenneth E Lipson, Yuri Nikolsky

**Affiliations:** 1 Pharmacology, 3M Pharmaceuticals, St Paul, Minnesota, USA; 2 GeneGo Inc, St. Joseph, Michigan, USA

## Abstract

**Background:**

Plasmacytoid Dendritic Cells (pDC) comprise approximately 0.2 to 0.8% of the blood mononuclear cells and are the primary type 1 interferon (IFN), producing cells, secreting high levels of IFN in response to viral infections. Plasmacytoid dendritic cells express predominantly TLRs 7 & 9, making them responsive to ssRNA and CpG DNA. The objective of this study was to evaluate the molecular and cellular processes altered upon stimulation of pDC with synthetic TLR 7 and TLR 7/8 agonists. To this end, we evaluated changes in global gene expression upon stimulation of 99.9% pure human pDC with the TLR7 selective agonists 3M-852A, and the TLR7/8 agonist 3M-011.

**Results:**

Global gene expression was evaluated using the Affymetrix U133A GeneChip^® ^and selected genes were confirmed using real time TaqMan^® ^RTPCR. The gene expression profiles of the two agonists were similar indicating that changes in gene expression were solely due to stimulation through TLR7. Type 1 interferons were among the highest induced genes and included IFNB and multiple IFNα subtypes, IFNα2, α5, α6, α8, α1/13, α10, α14, α16, α17, α21. A large number of chemokines and co-stimulatory molecules as well as the chemokine receptor CCR7 were increased in expression indicating maturation and change in the migratory ability of pDC. Induction of an antiviral state was shown by the expression of several IFN-inducible genes with known anti-viral activity. Further analysis of the data using the pathway analysis tool MetaCore gave insight into molecular and cellular processes impacted. The analysis revealed transcription networks that show increased expression of signaling components in TLR7 and TLR3 pathways, and the cytosolic anti-viral pathway regulated by RIG1 and MDA5, suggestive of optimization of an antiviral state targeted towards RNA viruses. The analysis also revealed increased expression of a network of genes important for protein ISGylation as well as an anti-apoptotic and pro-survival gene expression program.

**Conclusion:**

Thus this study demonstrates that as early as 4 hr post stimulation, synthetic TLR7 agonists induce a complex transcription network responsible for activating pDC for innate anti-viral immune responses with optimized responses towards RNA viruses, increased co-stimulatory capacity, and increased survival.

## Background

Dendritic cells constitute a heterogeneous population of antigen presenting cells that are critical for bridging the innate and the adaptive immune responses [1-3]. In the blood, DC can be sub-divided into two major populations, CD11c+ and CD11c-. The CD11c+ population is thought to be myeloid derived, therefore called myeloid or conventional DC (cDC). The CD11c- population, also known as plasmacytoid dendritic cells (pDC) appears to be lymphoid-derived and constitutes a population of cells that can migrate from the blood directly to lymphoid tissues [4]. pDC comprise approximately 0.2 to 0.8% of the blood mononuclear cells and are the primary type 1 interferon (IFN), producing cells, secreting high levels of these cytokines in response to viral infections [5] and stimulation with TLR7 and TLR9 agonists [6,7]. Type I IFNs are clinically important cytokines and are used for anti-viral [8] and anti-cancer therapy [9]. In addition to their direct anti-viral and anti-proliferative affects, type I interferons are important in bridging the innate and adaptive immune responses. Type 1 interferons have been shown to increase the expression of MHC class I and II, enhance co-stimulatory marker expression on DC, modulate immunoglobulin production, synergize with IL-12 to enhance IFN-γ production, and augmente NK and CTL responses [2,5,3].

pDC express a limited repertoire of toll like receptors (TLR), expressing predominantly TLR7 and TLR9 [10] and can be stimulated to produce large amounts of type 1 IFN in response to the natural TLR7 and TLR9 agonists guanosine- and uridine-rich ssRNA and DNA containing CpG motifs respectively [11,12]. Synthetic imidazoquinoline-like molecules exemplified by imiquimod (R-837) and resiquimod (R-848) have been identified as TLR7 agonists based on their inability to induce the cytokines TNFα, IL-12, or IFNα in TLR7-deficient mice [13]. In HEK293 cells transfected with TLR7 or TLR8, imiquimod and 3M-852A have been shown to posses a much higher potency at activating NFκB through TLR7 compared to TLR8, thus showing selectivity for TLR7 [14,15]. Resiquimod on the other hand activated NFκB through TLR7 and TLR8 at comparable levels [14] as did 3M-011 [16]. The TLR7 agonists imiquimod, and 3M-852A were found to activate pDC for the production of IFNα while the TLR8 agonist 3M-002 activated cDC for the production of IL12, and the TLR7/8 agonist resiquimod activated both cDC and pDC for cytokine production [14]. Thus, the TLR7 agonist 3M-852A is an ideal tool to study the molecular processes impacted through TLR7 in pDC.

The TLR7 agonist imiquimod is used for the treatment of various virally induced diseases such as genital warts [17] and for the treatment of cancerous and pre-cancerous skin lesions such as basal cell carcinoma (BCC) [18] and actinic keratosis (AK) [19]. The anti-viral and anti-tumor activity of synthetic TLR7 agonists has been attributed to the induction of cytokines such as IFNα [20,21]. Gene expression analysis of skin biopsies of patients with AK and BCC after topical treatment with imiquimod has shown that the TLR7 agonist induces the expression of a large number of interferon inducible genes indicating the importance of type 1 interferons in the mechanism of resolution of pre cancerous and cancerous skin lesions [22-24]. The TLR7 agonist 3M-852A is currently in phase II clinical trials for the therapy of cancer.

To date, the molecular pathways and processes of TLR7-mediated activation of pDC, the primary target of TLR7 agonists have not been thoroughly characterized. Therefore, the objective of this study was to evaluate the molecular and cellular pathways and processes impacted by stimulation of pDC with TLR7 agonists using global gene expression analysis in highly pure preparations of pDC. To this end, we evaluated global gene expression changes after stimulation of human pDC of greater than 99% purity with the TLR7 agonist 3M-852A, and the TLR7/8 agonist 3M-011 using Affymetrix U133A GeneChips. Further analysis of the data in the context of Gene Ontology classification and gene regulatory network analysis enabled us to identify molecular and cellular pathways impacted by stimulation of pDC through TLR7.

## Results and discussion

### Characterization of pDC Purity

**Figure 1:**
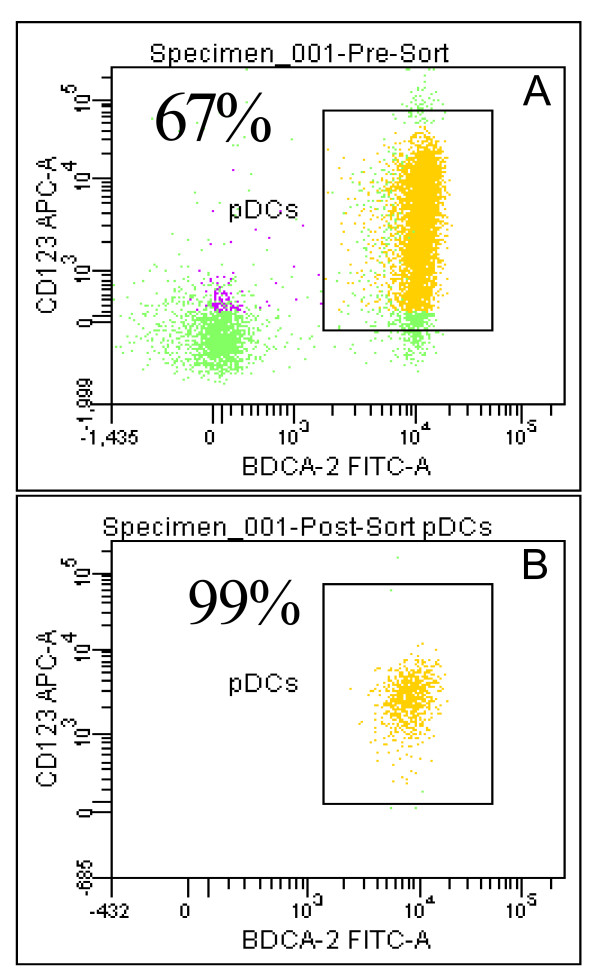
**Purity of pDC preparations as determined by flow cytometry**. Representative histograms for partially purified pDC (A) and flow purified pDC (B). pDC were separated from PBMC using anti BDCA4 microbeads. The partially purified cells were then labeled with CD123-APC, BDCA2-FITC, and CD11c-PE antibodies and sorted on a FACSAria flow cytometer to a final purity of > 99%. Numbers in the top, left hand corner of the dot plots indicate the percentage of CD123+, BDCA-2+, CD11c- cells.

Even though pDC are reported to express high levels of TLR7, other cells in PBMCs, such as monocytes also express TLR7 [10] and can contribute to TLR7-mediated events. Therefore, in order to determine TLR7-mediated gene expression specific to pDC, high purity pDC preparations are necessary. Positive cell selection using BDCA4 antibody resulted in pDC enriched preparations with variable purity ranging from 40% to 80%. Further purification using flow cytometry after staining cells with APC-labeled anti-CD123 antibody and FITC-labeled BDCA2 antibody resulted in pDC preparations that were greater than 99% pure. Figure 1A and 1B show representative histograms from flow cytometer analysis of pDC-enriched (67%) preparation (Figure 1A) and a 99% pure pDC preparation (Figure 1B) after flow purification, as evaluated by the percentage of CD123+, BDCA2+, CD11c- cells.

**Figure 2:**
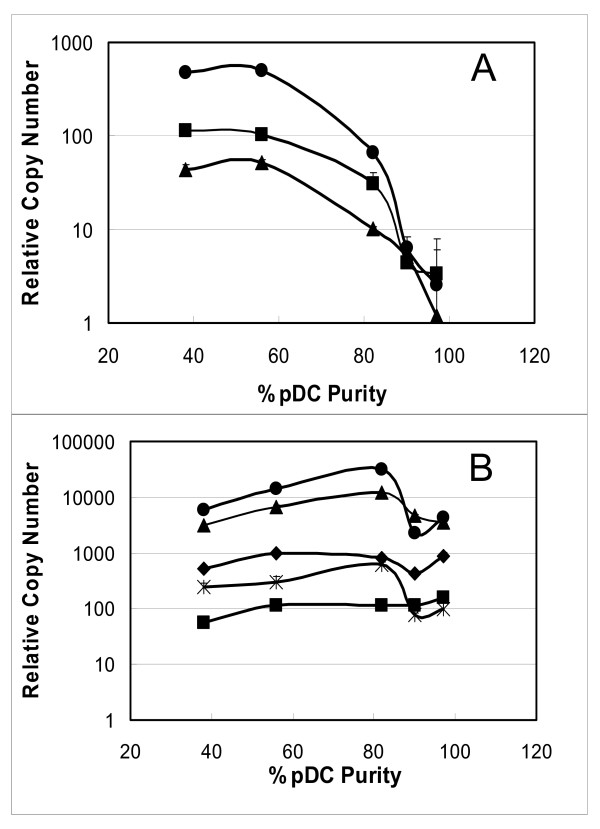
**Increasing purity of pDC preparations correlate with decrease in the expression of TLR4, TLR5, and TLR8**. (A) Change in expression of TLR4 (circles), TLR5 (squares), and TLR8 (triangles), and (B) TLR1 (diamonds), TLR6 (squares), TLR7 (triangles), TLR9 (circles), and TLR10 (stars) with increasing purity of pDC preparation. TLR expression was determined by real time RTPCR analysis. Relative copy number was calculated with respect to 2 ng cDNA after normalization to expression levels of GAPDH in each sample.

Purity of pDC preparations were also followed by monitoring the expression of various TLRs using real time RTPCR. Figure 2A and 2B show the expression of various TLRs in preparations of varying pDC purity. Figure 2A shows that as the purity of the pDC preparation increased, the expression of TLR4, TLR5 and TLR8 decreased. Conversely, the expression of TLR7, TLR9, and TLR10 increased up to a purity of 82% then decreased somewhat (Figure 2B), potentially indicating loss of a cell type which may be enriched in expression of these TLRs. Alternatively the variability in the expression of these TLRs for preparation > 82% pDC purity may be explained by donor-to-donor variability in expression. The expression of TLR1 and TLR6 remained somewhat constant.

### Induction of Type 1 Interferons genes: confirmation by real time RTPCR

GO molecular function analysis of Affymetrix GeneChip data showed that 14 out of 68 genes classified as hematopoeitin/interferon-class (D200-domain) cytokines, and 10 type-1 IFN genes were increased in expression by the TLR7 agonists. In addition, these genes were the highest induced genes in the data set with a median Log2 fold change in expression (across compounds and donors) ranging from 2.6 to 13. The interferon genes shown to be consistently induced in all donors were IFNA1, IFNA2, IFNA4, IFNA5, IFNA7, IFNA10, IFNA13, IFNA14, IFNA16, IFNA17, IFNA21, IFNB1, and IFNW1. IFNA6 and IFNA8 expression was donor variable, IFNA6 expression was detected in 3 of 4 donors for 3M-852A and 1 of 2 donors for 3M-011. IFNA8 expression was detected in 2 of 4 donors for 3M-852A and, 1 of 2 donors for 3M-011.

In order to confirm the induction of the interferon genes, we performed real time RTPCR. Analysis of the time dependence of induction of type 1 interferons by 3M-852A from 1 donor using real time RTPCR (data not shown) showed that induction of IFNB, IFNA2, IFNA8 and IFNA14 was high (log2 fold change = 6.6) at 30 minutes post stimulation and reached peak levels at 1.5 hr (log2 fold change ~12), and remained at high levels 4 hr post stimulation. Induction of IFNA5, IFNA6, IFNA10, IFNA13, and IFNA21 were relatively low (log2 fold change of 2 to 5) 30 minutes post stimulation and peak levels were achieved 4 hrs post stimulation. Table 1 shows a comparison of expression data obtained by Affymetrix analysis and real time RTPCR analysis at 4 hr post stimulation for the interferon genes as well as for other selected genes. Specific primers and probes for RTPCR were not available for four of the interferon alpha subtypes (IFNA4, IFNA7, IFNA16, and IFNA17) at the time of data collection. The reagents for IFNA1 in the RTPCR study detected both IFNA1 and IFNA13. Of the interferon genes tested by both Affymetrix and real Time RTPCR, all IFNA subtypes and IFNB showed increased expression by both methods indicating good concordance.

**Table 1:** Comparison of gene expression changes as determined by Affymetrix GeneChip analysis and real time RTPCR

Gene Symbol	Log2 Fold Change: Affymetrix		Log2 Fold Change: Real Time RTPCR	
	3M-852A	3M-011	3M-852A	3M-011

IFNA1	7.2	10.2	8.9	8.0
IFNA10	5.4	6.7	7.5	7.0
IFNA13	5.5	6.6	NA	NA
IFNA14	6.7	7.9	10.2	8.2
IFNA16	6.0	5.0	NA	NA
IFNA17	5.1	4.3	NA	NA
IFNA2	6.8	6.0	11.0	9.3
IFNA21	7.7	6.7	9.2	8.0
IFNA4	5.9	5.1	NA	NA
IFNA5	6.3	5.8	9.8	8.6
IFNA6	2.6	1.3	5.0	3.7
IFNA7	5.9	4.9	NA	NA
IFNA8	2.8	2.7	8.0	7.2
IFNB1	8.0	6.2	10.4	8.6
Bcl-2	1.4	1.2	3.1	2.7
BCL2L1	4.2	3.8	5.1	5.0
CCL3	2.6	1.2	3.2	0.9
CD80	2.5	1.8	5.6	4.9
CXCL10	5.2	5.7	7.5	8.3
GIP2	2.8	3.2	2.0	4.0
MX1	1.4	1.6	2.3	2.4
MYC	4.6	2.2	7.1	5.9
MYD88	1.3	1.6	2.1	2.3
PTGS2	3.5	1.2	5.4	3.1
STAT1	1.0	1.2	1.3	1.1
TNF	2.4	1.4	3.2	2.2

^1^Taqman primers and probes for IFNA1 in the real time RTPCR detect both FINA1 and IFNA13. Fold change in expression were calculated with respect to un-stimulated pDC. For the Affymetrix data, values are averages of data sets for four donors for 3M-852A and two donors for 3M-011. For the real time RTPCR data, values are averages of two data sets from two donors for each compound. NA designates not analyzed.

In humans, the type 1 interferon family of genes consists of 13 IFNA subtypes, IFNB, IFNW, IFNE1 also know as IFN-tau1, and IFNK [25]. pDC are known to produce high levels of type 1 interferons in response to viruses [1,26,27] and various toll like-receptor ligands including TLR7[14] and TLR9 [28]. We show that stimulation of pDC through TLR7 results in the induction of IFNB and the majority (if not all) IFNA subtypes induced by viruses such as herpes simplex virus type 1(HSV-1) and Sendai virus (SV) [26] and H3N2 flu virus [27]. Since the anti-viral and anti-tumor activities of the different IFNA subtypes have been shown to differ [29-31] induction of a variety of IFNA subtypes by TLR7 agonists may be an advantage for anti-viral and anti-tumor therapy.

### TLR7-mediated cytokine protein expression

**Figure 3:**
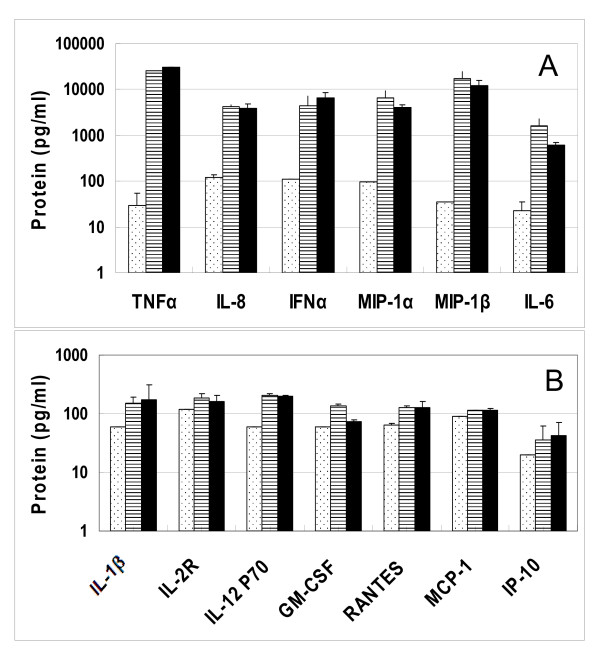
**Cytokines secreted by flow purified pDC 4 hr post-stimulation with TLR7 agonists**. Cell culture supernatants collected after stimulation were evaluated for cytokine protein expression using a Luminex 25-Plex assay. (A) Secreted TNFα, IL8, IFNα, MIP-1α, MIP-1β and IL6. (B) secreted IL1β, IL2R, IL12P70, Rantes, GM-CSF, MCP-1, and IP-10. Dotted bars, vehicle stimulated samples; hatched bars, 3M-852A-stimulated samples and solid bars, 3M-011-stimulated samples. The results are expressed as mean + SD, n = 2 donors.

In order to confirm the expression of some of the genes at the protein level, ELISA and Luminex multiplex protein assays were used to determine the expression of several secreted cytokines and chemokines. Supernatants were collected after stimulation of pDC for 4 hr. Of the 25 cytokines and chemokines tested, IFNα, TNFα, IL8, IL6, MIP-1α (CCL3), and MIP-1β (CCL4) proteins were induced at high levels > 1000 pg/ml. This is consistent with the high level of expression of these genes observed by Affymetrix and real time RTPCR analysis (Table 1). Figure 3A shows the induction of these cytokines for 3M-852A and 3M-011. In contrast, low levels of IL-1β, IL12p70, GM-CSF, Rantes (CCL5), MCP-1 (CCL2), and IP10 (CXCL10) proteins were detected (Figure 3B). The genes for IL1β, IL12, GM-CSF, and MCP1 (CCL2) were not increased in expression in the Affymetrix or real time RTPCR data. In contrast, high levels of gene expression (up to a fold change of 900) were observed for CXCL10 and CCL5. The seeming disparity may be due to the fact that data was collected at 4 hr post stimulation and the proteins for these genes may not have reached peak expression. Consistent with the gene expression data, the proteins IL-1RA, IL-2, IL-4, IL-5, IL-7, IL-10, IL-13, IL-15, IL17, IFNγ, MIG (CXCL9), and Eotaxin (CCL11) were not induced by TLR7 agonists in pDC 4 hr post stimulation. However, induction of these genes and proteins at a different time than 4 hr post stimulation can not be ruled out.

### GO classification of Affymetrix GeneChip data

**Figure 4:**
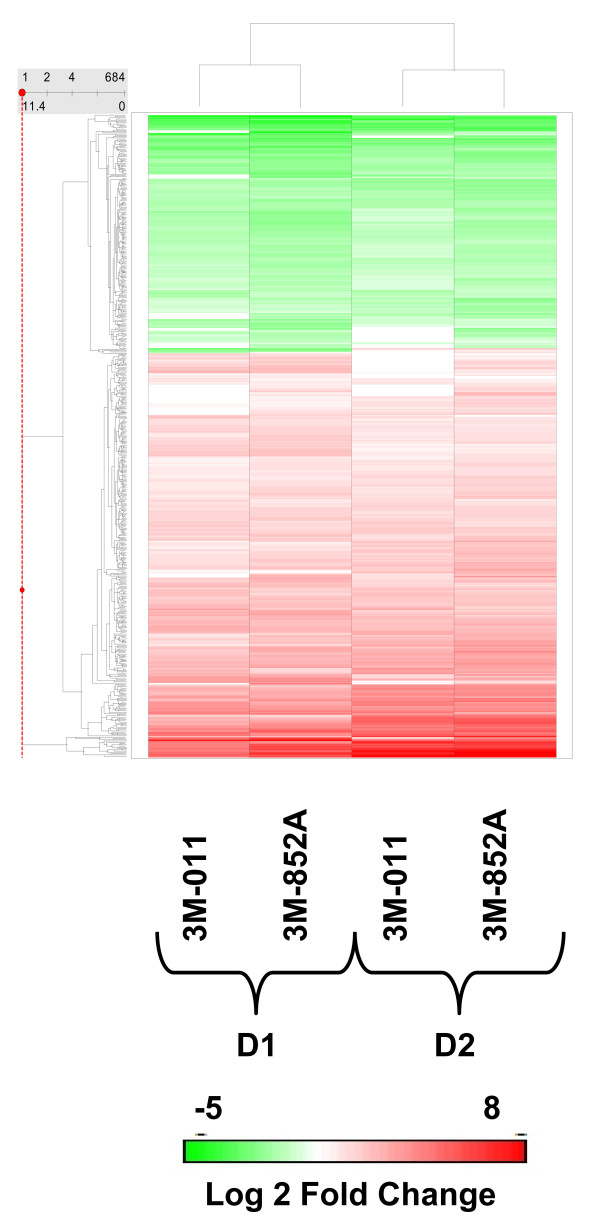
**Cluster analysis of 680 genes regulated in expression by 3M-852A and 3M-011**. Gene expression analysis was performed at 4 hr post-stimulation of flow-purified pDC with 3M-852A and 3M-011 using the Affymetrix GeneChip U133A as described in Methods. D1 and D2, designate two different donors for pDC preparation in which the pDC purity was > 99%. Global gene expression was determined using the Affymetrix U133A GeneChip. Two-way hierarchical clustering was performed as described in the Methods section, using the Unweighted Pair-Group Method with Arithmetic mean (UPGMA) and the Euclidean similarity measure. The log2 fold change values were used for the analysis. Insert bar chart shows the expression change scale with red, green, and white signifying increased, decreased, and unchanged expression, respectively. Expression changes were evaluated with respect to vehicle stimulated pDC from the same donor. Expression changes for the 680 genes are documented in Additional File 1.

In order to determine TLR7-mediated global gene expression changes, Affymetrix GeneChip analysis was performed after stimulation of pDC with TLR7 selective agonists 3M-852A, and the TLR7/8 agonist 3M-011. Stimulation of pDC for 4 hours resulted in changes in expression of 680 genes with consistent expression changes across replicates for at least one of the compounds tested (see Additional File 1). Out of the 680 genes, 430 genes were increased in expression and 250 genes were decreased in expression due to stimulation with at least one TLR agonist in all donors tested. Figure 4 shows a two way hierarchical clustering of the 680 genes altered in expression. Samples clustered primarily according to donor rather than compound indicating that the compounds exhibit an overall similar gene expression profile, consistent with gene expression changes regulated through activation of TLR7. Subtle differences are apparent in the response of the same donor to the different agonists, primarily in the magnitude of change in expression.

In order to explore the biological significance of TLR7-mediated gene expression changes, we analyzed their Gene Ontology (GO) classification using MetaCore as well as the GO browser in Spotfire for Functional Genomics. The most highly represented processes include: programmed cell death, specifically negative regulation of programmed cell death and anti-apoptosis, immune response, response to virus, inflammatory response and defense response. Analysis for GO function classification showed that the most highly represented GO function is cytokine activity, including hematopoeitin/interferon class (D200 domain) containing proteins and chemokines (p values 10-^11 ^to 10^-5^). Other functional groups enriched in the data set include genes with oxidoreductase activity, protein ubiquitin ligase activity and guanine nucleotide binding activity. The role of some of these processes in pDC activation will be discussed below.

### Analysis of gene regulatory networks

In order to further understand the molecular processes initiated following stimulation of pDC with 3M-852A and 3M-011 the expression data were input into MetaCore and analyzed for protein regulatory networks enriched with the expression data. The 680 genes altered in expression were used as a list of input nodes and subjected to the Analyze Networks (Receptors) algorithm and the Analyze Networks (Transcription regulation) algorithm (see Methods for details). These algorithms generate a virtual shortest paths network between all nodes from the input list and parse them into sub-networks centered on receptors and transcription factors respectively. Analysis of the data using the receptor networks algorithm resulted in 30 sub networks focused on various receptors leading to activation of various transcription factors. The top ten significant networks were composed of 40 to 107 genes altered in expression with p value ranging from 6e^-66 ^to 4e^-128^. These top ten networks included transcription regulation initiated through activation of the chemokine receptor CCR3, insulin receptor, IGF-1 receptor, IFNα/β receptor, and CD80-CD86/CTLA4 system.

Analysis of the gene expression data in MetaCore using the transcription regulation algorithm resulted in 84 sub networks centered on various transcription factors. The top 10 networks consisted of transcription factors which directly regulated the expression of 12 to 37 genes, with a total network size of 50 to 80 genes and p value for significance of network ranging from 8e^-95 ^to 8e^-128^. The top ten networks were for the transcription factors p53, CREB1, IRF1, RelA (p65), HNF6, IRF2, c-Rel, IRF8, NFkb2, and NFkb p50/p65. These networks were enriched in genes with GO ontology classification of regulation of physiological processes and regulation of apoptosis (CREB1, p53, NFkb, c-Rel) and anti-bacterial/antiviral humoral responses (IRF1, RelA, HNF6, IRF8, NFkB2, NFkB p50/p65). Two other networks centered on IRF7 (p value = 6e^-85^) and IRF9 (p value = 1e^-81^) and were enriched in genes with GO ontology classification of defense response and virus response. Some of the interaction networks derived from the above analysis will be discussed in the following sections.

### Transcription regulatory network indicates utilization of various transcription factors following activation of TLR7

**Figure 5:**
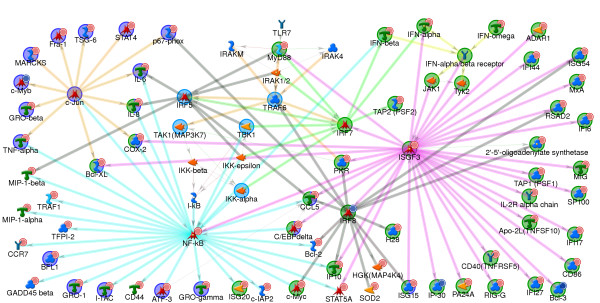
**Transcription regulatory network indicates utilization of various transcription factors following activation of TLR7**. Protein-interaction network was generated for expression changes in pDC stimulated with 3M-852A and 3M-011 4 hr post-stimulation. The network shows genes altered in expression in the data set which were transcriptionally regulated via IRF7, NFkB, cJun, IRF5, IRF8 through potential interaction with the TLR7 pathway and through ISGF3 via activation of the type 1 interferon receptor. Network was generated using the Analyze network (receptor) and the direct interactions algorithms using the whole expression data. Major hubs have been highlighted in bold. Remaining details of network are as described in Methods. Legend describing symbols in the network is found in Additional file 2.

Recent research has implicated the involvement of various transcription factors including NFkB, AP1, IRF7, and IRF5 in signaling through TLR7 [32] in various immune cells as well as cells transfected with TLR7 [14]. Even though the direct activation of IRF7 through TLR7 has been demonstrated in pDC, the direct activation of other transcription factors has not been documented. Figure 5 shows a transcription network which illustrates interactions between the TLR7 signaling pathway components and transcription factors known to be activated by the TLR7 pathway in other cell types including IRF7, NFkB, IRF5, and c-Jun. The network includes protein-protein binding interactions, protein phosphorylation and transcription regulation. Some of the TLR7 signaling components have been omitted for the sake of clarity. The figure shows genes that are know to be transcriptionally regulated by IRF7, NFkB, IRF5, IRF8, c-Jun and through ISGF3 via activation of the IFNα/β receptor. Exemplified are transcriptional regulation through NFkB, e.g. CCR7, Gro-1 (CXCL1), Gro-gamma (CXCL3) and CD44; through IRF7: e.g. type 1 interferons and TAP2; through NFkB and IRF5, e.g. IL8 and MIP-1β (CCL4); through NFkB and c-Jun, e.g. TNFA, MIP-β, Gro-β (CXCL2); through IRF8, H28 and HGK (MAP4K4); through NFkB, IRF5 and ISGF3, e.g. CCL5; ISG20 and BCL2.

Interferon regulatory factor 8, IRF8 (also known as ISCBP8) is a transcription factor recently identified as being important for the development of type I interferon-producing cells (pDC) in the mouse [33]. IRF8-/- mice have also been shown to be unresponsive to CpG in induction of cytokines, thus indicating the importance of IRF8 in TLR9 signaling [34]. In addition, interactions between IRF8 and TRAF6 resulting in enhanced ubiquitination of TRAF6 has been reported in macrophages [35]. Whereas the role of IRF7 in the signaling ofTLR7 has been established, the role of IRF8 in signaling of TLR7 has not been elucidated. Upon stimulation of pDC with 3M-852A the expression of IRF8 decreased by 2 to 4 fold. The significance of the decrease is not clear but may be related to feed-back regulation.

Interaction of the type 1 interferons with the interferon receptor results in activation of the JAK-STAT pathway culminating in the translocation of transcriptional complex ISGF3 composed of STAT1, STAT2 and IRF9 (ISGF3) or STAT1 dimers (AAF, IFN-a activated factor) to the nucleus [36]. The complexes bind to specific DNA sequences called IFN-α stimulated response elements (ISRE) found in the promoter regions of target genes resulting in increased expression of several hundred genes. All three components of the ISGF3 complex, IRF9, STAT1 and STAT2 were increased in expression upon stimulation of pDC with TLR7 agonists.

A large number of genes transcriptionally regulated through ISGF3 are increased in expression (see Additional File 1). A small number of these genes are shown in the interaction network of Figure 5, including, MXA, OAS1, TNFSF10, IFI44, ISG56 and NMI. Many of the genes that are inducible by type 1 interferons can also be transcriptionally regulated directly via other transcription factors. For example, the expression of CD86, MIG (CXCL9), IP10 (CXCL10) and CCL5 is known to be directly regulated via NFkB and IRF5 [37-40] as well as indirectly through type 1 interferons.

Thus, the data demonstrate the complex network of transcriptional regulation initiated through activation of pDC through TLR7. Direct TLR7-initiated transcription regulation through IRF7, has been documented in pDC [41]. The dependence of TLR9-mediated IL6 induction and pDC survival on NFKB1 and c-Rel has also been documented [42]. TLR7-mediated transcription regulation via IRF5, NFkB and AP1 has not been demonstrated in pDC but is well documented in other immune cells. Further study is required to demonstrate the direct utilization of these transcription factors in TLR7 mediated activation of pDC.

**Figure 6:**
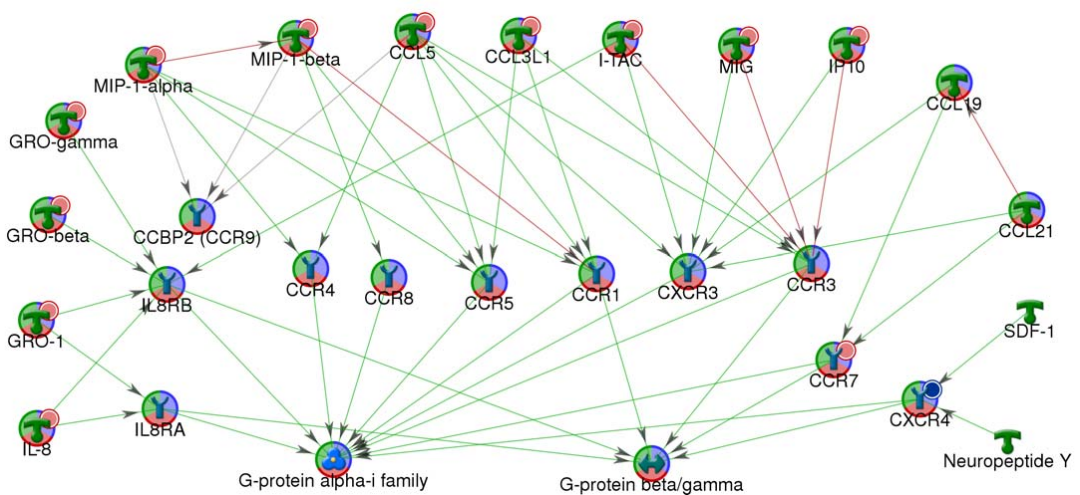
**Stimulation of pDC with 3M-852A and 3M-011 results in increased expression of a large number of chemokines**. Protein-interaction network was generated for expression changes in pDC 4 hr post stimulation with 3M-852A and 3M-011. The network was generated using a list of genes in the GO function classification of chemokines and the shortest path algorithm. The network summarizes interactions between chemokines increased in expression and their respective receptors. Green arrows indicate binding interactions or covalent modifications that result in activation or increased transcription. Red arrows indicate binding interactions or covalent modifications that result in inhibition of activity or suppression of transcription.

**Figure 7:**
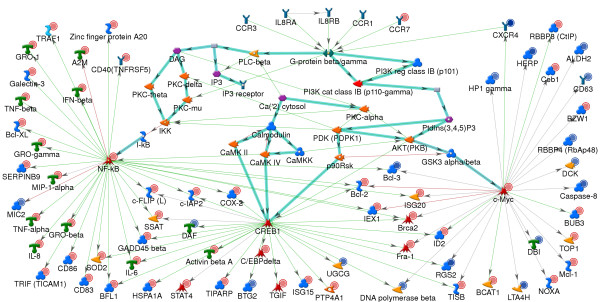
**Potential transcription regulation of genes through pathways secondary to stimulation of TLR7**. Protein-interaction network was generated for expression changes in pDC stimulated with 3M-852A and 3M-011 4 hr post stimulation. Network was generated using the Analyze network (receptor) and the direct interactions algorithms using the whole expression data. The network shows genes altered in expression in the data set that are transcriptionally regulated through NFkB, c-Myc, and CREB1. Potential activation of these transcription factors through the chemokine receptor CCR1, CCR3 and the IL8 receptors is depicted. Bold green lines highlight the canonical pathways for activation of the three transcription factors. Fine lines going out of the transcription factors designate transcription regulation. Remaining network details are as described in Figure 6.

In addition to the genes regulated by the transcription factors illustrated in Figure 5, analysis in MetaCore showed that a large number of genes that are increased in expression are known to be regulated by the transcription factors CREB1, c-Myc and p53. Four hr. post-stimulation with TLR7 agonists, one would expect transcriptional regulation through other secondary pathways activated by the genes induced in the primary TLR7-mediated pathways. In addition to type 1 interferons, a large number of other cytokine and chemokine genes were increased in expression including the chemokines, CCL5, IL6, IL8, MIP1A, MIP1B, and the cytokines TNFA and TNFB. In fact, at 4 hr post-stimulation, significant amounts the proteins for IL6, IL8, TNFα, MIP-1α and, MIP-1β were produced. These cytokines can act in an autocrine fashion to activate the IL8 receptors, CCR1 and CCR3, which are linked to G proteins that can in turn activate various transcription factors. Figure 6 shows a protein interaction network illustrating the various chemokines induced in pDC and their respective G-protein-coupled receptors. Potential activation of the transcription factors CREB1, c-Myc and NFkB via the chemokine receptors CCR1, CCR3 and the IL8 receptors is depicted in Figure 7. Chemokine receptors activate various guanine nucleotide proteins (G proteins) including the beta/gamma subunits which result in the activation of the phospholipase beta (PLCB) and Phosphatidyl inositol kinase (PI3K) pathways [43,44]. These signaling pathways culminate in the activation of various transcription factors including CREB, cMyc, and NFkB. In addition to the chemokines for these receptors, one of the G-proteins, GNG11 (guanine nucleotide binding protein gamma 11) is also increased in expression.

**Figure 8:**
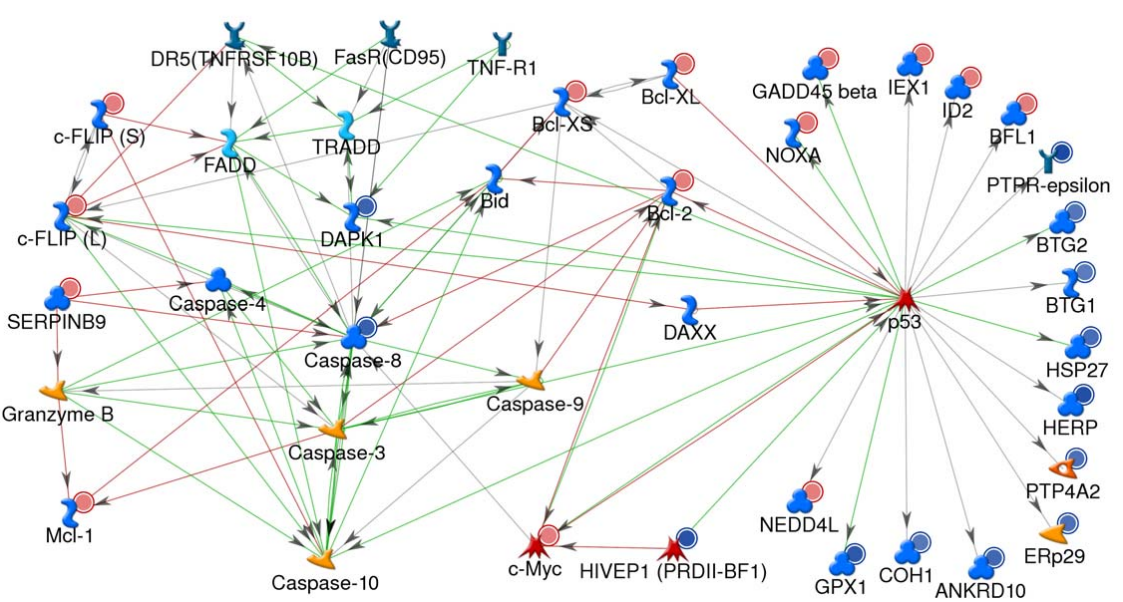
**Stimulation of pDC with 3M-852A and 3M-011 results in the induction of an anti-apoptotic gene expression program**. Protein-interaction network was generated for expression changes in pDC 4 hr post stimulation with 3M-852A and 3M-011. The network was generated from a list of genes with GO process classification of anti-apoptosis, using the shortest path algorithm. The network highlights the large number of anti-apoptotic genes that are increased in expression with a concomitant decrease in expression of key pro-apoptotic genes including several that are transscriptionally regulated by p53. Remaining network details are as described in Figure 6.

The transcription factors CREB1, c-Myc and NFkB regulate the transcription of genes important in cell proliferation, differentiation, and apoptosis. Whereas most of the genes regulated by NFkB and CREB1 are increased in expression, a significant percentage of those regulated by c-Myc are decreased in expression. Several of the down regulated genes are involved in the regulation of cell cycle and of apoptosis and include Caspase 8 and BTG2 [45,46]. Genes increased in expression include pro-inflammatory genes, genes with growth factor activity and or proliferative activity including GRO1, GROG, GROB, MCL-1 [47,48] and anti-apoptotic activity, BCL2, c-IAP2, CFLIP, SERPINB9 [49-51]. Thus, the transcription regulatory network shown in Figure 7 suggests that 4 hr post stimulation with TLR7 agonists, pDC are programmed for cell survival. This is shown more clearly in Figure 8; a protein regulatory network built using a list of genes with GO classification of anti-apoptosis as the input nodes.

### Induction of an anti-apoptotic gene expression program

Activation of pDC through TLR7 agonists results in the induction of TNFR-associated genes that are known to induce apoptosis. These include TNFA, TNFB and TNFSF10 (TRAIL). The protein products of these genes may be expected to induce apoptosis in pDC via autocrine regulation. However, the gene expression data shows that TLR7 agonists induce an anti-apoptotic and pro-survival gene expression program in pDC. Figure 8 depicts a protein regulatory network for TNFR1, TNFRSF6 (FasR) and TNFRSF10 (TRAIL receptor)-mediated apoptosis. TNFR ligands such as TNFA, TRAIL and FAS ligand exert their apoptotic effect through activation of the caspase cascade which results in the degradation of several proteins critical to cell survival pathways [45]. Caspase 8 (CASP8) and DAPK1 (death-associated protein kinase) genes central to TNFR1, FasR and TNFRSF10 (TRAIL receptor) mediated apoptotic death [52] are decreased in expression Concomitant with the decreased expression of apoptosis mediators, several inhibitors of activation of caspases and activators of pro-survival genes are increased in expression. The anti-apoptotic gene BCL2 which is increased in expression, inhibits apoptosis mediated by TNFR1 and caspase 8 [49,53] and is in turn activated by pro-survival genes such as beta-catenin. The protein products of CFLAR, c-FLIPS and c-FLIPL inhibit the death receptor adaptor molecules FADD, DAXX and caspase 10 [50,54,55]. Other anti-apoptotic genes increased in expression by TLR7 agonists in pDC include the pro-survival gene MCL-1, which is tightly regulated by pro-apoptotic genes such as Caspase 3 and Granzyme B [48,56]; SERPINB9 which inhibits Caspase 1 and Granzyme B [51,57]; cIAP2 (BIRC3, [not shown in figure) which promotes degradation of caspase 3 and caspase 9 through its ubiquitin ligase activity [58,59]; and BCLXL (BCL2L1) which inhibits activation of the tumor suppressor protein p53 [60]. Other pro-survival genes increased in expression include Pim-2 and TPL2 (not shown in network). Pim-2 (pim-2 oncogene) inhibits the pro-apoptotic protein BAD (BCL2 antagonist of cell death) [61] and activates the pro-survival gene TPL2 (MAP3K8) [62]. Figure 8 also shows that a number of genes that are positively regulated by the transcription factor p53 are decreased in expression, suggesting that transcription from p53 may be suppressed. Taken together, the gene expression data suggest that activation of pDC by TLR7 agonists induces an anti-apoptotic and pro-survival gene expression program, a state necessary for the migration of pDC to inflammatory sites and to lymph nodes.

### Interferon-inducible genes: Antiviral response

Interferon-inducible genes regulate cellular processes such as cell growth and differentiation, cell death, and T-cell costimulation, activation, and migration. Many of these genes also have direct antiviral activity, including MX1 (MXA) [63] OAS1 [64], PKR (EIF2AK2) [65], ISG15 (G1P2), RSAD2 (Cig5), [66], ISG56 [67], PLSCR1 [68], IRF1, IRF9, GBP1, IFI-6-16 and IFI27 [69]. Genes such as IFIH1 and IFI16 have been shown to regulate cell growth and differentiation [70]. The biological function of other interferon-inducible genes such as IFIT1, IFIT2, IFIT3, IFTM1, IFI35, G1P3, and ISG20 are not well understood. However, these genes have been shown to be part of the innate anti-viral response [71,72,69]. A comprehensive discussion of the biological role of IFN-inducible genes is beyond the scope of this manuscript. The few genes cited here demonstrate the important role that TLR7 plays in antiviral, anti-tumor, and immune-regulatory functions in pDC.

### Protein regulatory network indicates importance of ISGylation pathway in TLR7-mediated activation of pDC

One of the interferon-inducible genes increased in expression upon treatment of pDC with TLR7 agonists is ISG15, interferon stimulated protein 15 (G1P2, UCRP). ISG15 has been shown to function as a cytokine which stimulates IFNγ production as well as proliferation of natural killer cells and their cytolytic activity [73]. ISG15 contains two ubiquitin-like domains and is also know to be conjugated to various protein targets [74-76]. The protein ISGylation pathway uses a similar set of enzymes to that used by the ubiquitin pathway including the ubiquitin E1 like enzyme UBE1L [77], the ubiquitin E2 enzyme UBCH8 [78,79] the HECT type ubiquitin E3 ligase HERC5 (also known as ceb1) [80,81] and the ubiquitin E3 liagase EFP also known as TRIM25 [82].

**Figure 9:**
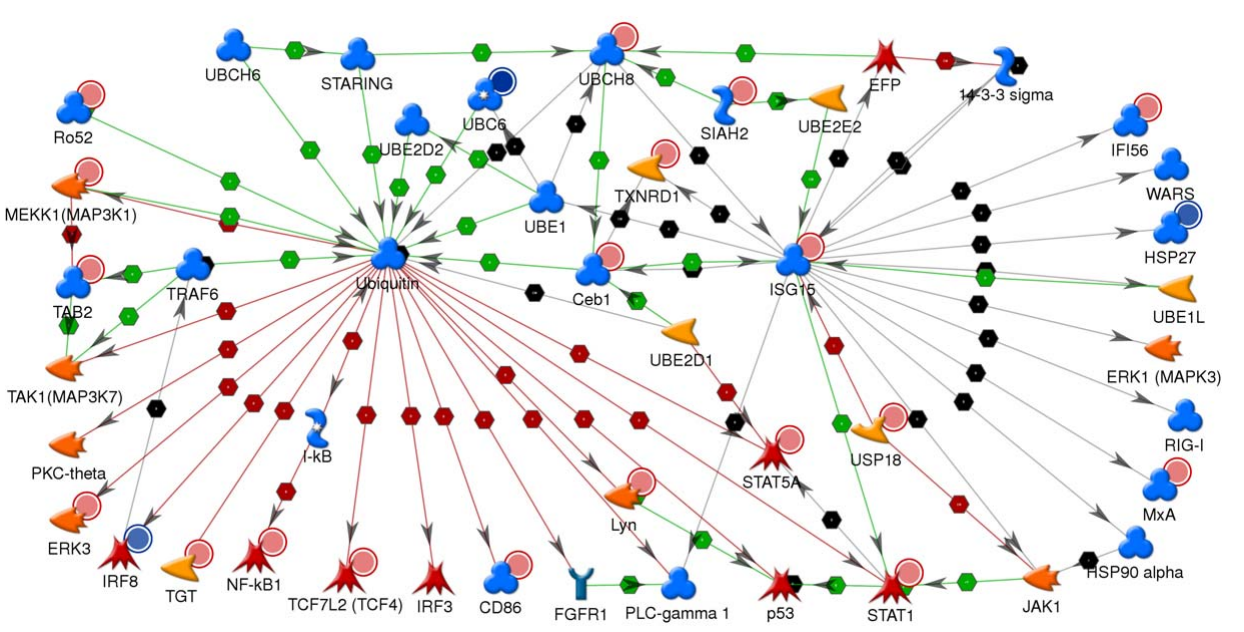
**Protein regulatory network indicates importance of ISGylation pathway in TLR7-mediated activation of pDC**. Protein-interaction network was generated for expression changes in pDC stimulated with 3M-852A and 3M-011 4 hr post-stimulation. The network was generated from a group of genes with the GO ontology classification of ubiquitin cycle using the shortest path algorithm. The network summarizes interactions between the ubiquitin and the ISG15 pathways for protein modification and the genes that are altered in expression upon stimulation of pDC with TLR7 agonists. Remaining network details are as described in Figure 6.

In this study, evidence for the importance of protein ISGylation following stimulation of pDC with TLR7 agonists is provided by increased expression of ISG15 and genes important in the ISGylation pathway. Analysis of GO process classification showed that 33 genes involved in the ubiquitin cycle were altered in expression upon treatment of pDC with 3M-852A and 3M011. Figure 9 exhibits a protein regulatory network built from this list of genes using the shortest path algorithm, and shows the interactions between the ubiquitin pathway leading to proteasome-dependent protein degradation and the protein ISGylation pathway. Shown in the interaction network are enzymes common to the ubiquitin and the ISGylation pathways UBCH8, HERC5 (ceb1) and EFP and a few of the numerous proteins that are ubiquitinated for protein degradation and those that are ISGylated. Notable proteins marked for degradation by ubiquitination are those relevant to the immune system such as IRF3, IRF8, STAT1, and STAT5. [83-86] Among the proteins known to be ISGylated are the ubiquitin enzymes UBCH8 and HERC5 [79,76], the anti-viral interferon-inducible genes PKR (EIF2AK2), MX1, and RIG1 [87], signaling proteins in the JAK-STAT pathway including JAK1, ERK1 and STAT1 [74,88] and IRF3 [89], which plays a major role in the innate anti-viral response. Evidence for cross regulation of the ubiquitin and ISGylation pathways has been reported. ISG15 has been shown to inhibit the ubiquitin/26S proteasome pathway [90,76], thus inhibiting the degradation of proteins such as IRF3 [89]. ISGylation of the ubiquitin E2 enzyme UBC13 (UBE2N) has also been shown to disrupt modification of this enzyme by ubiquitin [87].

ISG15 as well as several of the genes which are known to be ISGylated including UBCH8, HERC5 and USP18, and the innate anti-viral response genes RIG1, MX1, PKR, and IRF3 are also known to be inducible by type 1 interferons and viruses [66,79,90,91]. Experiments in ISG15 knockout mice have shown that IFN-induced antiviral responses of ISG15^-/- ^mice to vesicular stomatitis and lymphocytic choriomeningitis virus were not significantly altered [92]. In contrast, similar experiments show increased susceptibility towards influenza virus, herpes virus type 1 (HSV-1), and Sindbis viruses [66]. The difference in the antiviral activity of ISG15 towards the different viruses is not clear. It is postulated that the antiviral effectiveness ISG15 may depend on the effectiveness of mechanisms the particular virus may have evolved to counteract it. For example, influenza B virus produces a viral protein, NS1b which is capable of binding to ISG15 to prevent protein ISGylation [93]. Similar mechanisms in vesicular stomatitis and lymphocytic choriomeningitis virus may function to strongly counteract the antiviral effects of ISG15 as seen the work of Osiak et al [92]. The increased expression of enzymes important for ISGylation as well as innate antiviral genes that are targets of ISGylation thus underscores the importance of protein ISGylation in the innate, anti-viral response of pDC stimulated with TLR7 agonists.

### Change in expression of chemokines and chemokine receptors

The gene expression analysis in this study shows that activation of pDC results in increased expression of a large number of other cytokines and chemokines in addition to type 1 interferons (see Additional File 1). Figure 6 shows a protein interaction network for several CC and CXC chemokines induced by TLR7 agonists in pDC. The highest induced chemokines are ligands for CXCR3 with expression changes ranging from 20-fold to 120-fold. The CXCR3 ligands include CCL5 (Rantes) which is chemotactic for Memory T cells and monocytes, CXCL11, (ITAC) and CXCL9 (MIG) which are chemotactic for activated T cells, and the pleotrophic chemokine CXCL10 (IP10) which stimulates monocytes, natural killer (NK) cells and T cells, and modulates their migration [47]. In addition to CXCR3, CCL5 also activates the chemokine receptors CCR1, CCR3, CCR4, and CCR5. The ligands for the chemokine receptors CXCR1 (IL8RA) and CXCR2 (IL8RB); IL8, CXCL1 (GRO1), CXCL2 (GRO-beta), and CXCL3 (GRO-gamma) are chemotactic for a number of cell types including T cells, B cells, monocytes, macrophages, neutrophils and mast cells [47]. These chemokines are induced to a lower degree (2- to 12-fold change in expression) than the CXCR3 ligands CXCL9, CXCL10 and CXCL11. Thus, the data suggest that TLR7-activated pDC predominantly influence migration of activated T cells.

Only two chemokine receptors are altered in expression upon activation of pDC by TLR7 agonists; CXCR4 which is deceased in expression and CCR7, which is increased in expression. CXCR4 is the receptor for CXCL12 (SDF1, stromal cell-derived factor which is expressed by HEV (endothelial venules), bone marrow stromal cells and tumor stromal cells [94,95]. In contrast, the ligands for CCR7; CCL19 and CCL21 are expressed by HEV as well as stromal cells within T cell-rich areas [96]. In addition, increased expression of CCR7 on dendritic cells is critical for migration to lymph nodes [97,98]. Thus, the increased expression of CCR7 with the concomitant decrease in expression of CXCR4 more specifically predisposes TLR7 activated pDC to migrate to lymph nodes. It is interesting to note that CXCR4 is one of the receptors for entrance of HIV-1 into pDC [99]. The reduced expression of CXCR4 may thus have a dual role, in pDC migration and as an anti-viral mechanism.

### Activation of pDC for antigen processing and presentation and T-cell co-stimulation

Activation of pDC by TLR7 agonists alters the expression of various genes involved in antigen processing and presentation (see Additional File 1). TAP1 and TAP2, antigen peptide transporter 1 and 2 were moderately increased in expression. Genes involved in antigen presentation were also increased in expression, including a low and donor dependent increase in HLADQA1 (MHCII molecule) and a stronger increase in LAMP3 (DCLAMP). Various genes involved in T cell costimulation were also increased in expression including CD80, CD83, CD86 and CD40. In addition, TNFRSF9 (CD137, 4-1BB) and its ligand TNFSF9 (4-1BB-L) were increased in expression. TNFSF9 expressed on dendritic cells is known to interact with TNFRSF9 on activated T cells resulting in Th1 polarization and, T, and NK cell survival and expansion [100,101]. It is interesting that both the receptor and ligand-pair are increased in expression upon stimulation of pDC with TLR7 agonists, suggesting a role for this pair of molecules in pDC signal transduction. Thus, as early as 4 hr post-stimulation with TLR7 agonists, pDC are primed for antigen processing and presentation as well as co-stimulation of cells of the adaptive immune system.

### Activation of pDC through TLR7 leads to increased expression of genes in innate anti-viral pathways which respond to RNA viruses

Regulation of expression of type 1 interferons through TLR7 is believed to involve IRF7 and the adaptor molecule MyD88 [102,36]. In this study we show that stimulation of pDC with TLR7 agonists results in increased expression of genes known to be involved in TLR7-signaling and/or in signaling through other TLRs. The TLR7 adaptor molecule MyD88, TAB2 (TAK1-binding protein 2) and NFκB [103] are increased in expression. In addition, genes that interact with TRAF6, the central mediator of signaling through several TLRs; such as MAP3K8 (TPL2), and TNFAIP3 (zinc finger protein A20) were also increased in expression. MAP3K8 has been shown to interact with TRAF6, IKK-alpha and NFκB [104] and is required for TLR-mediated ERK activation and induction of pro-inflammatory cytokines [105]. TNFAIP3 is now recognized as a negative regulator of TLR signaling [106] as well as signaling through the cytosolic anti-viral pathway mediated by RIG1 [107]. RIPK2 (receptor interacting protein kinase 2) which activates IKK-alpha (inhibitor of nuclear kappa-B kinase alpha) resulting in IRF7-mediated induction of IFNA [108] and NFκB-mediated induction of chemokines [109] was also increased in expression. Thus, activation of TLR7 by TLR7 agonists results in increased expression of signaling components of TLR7 as well as other TLRs, thus potentially amplifying TLR signaling pathways. While some genes are positive drivers of the signaling pathways others are negative regulators of the pathways, indicating tight regulation of these signals.

In addition to known components of TLR7 signaling, genes in the signaling pathways for TLR3 and the cytosolic innate anti-viral pathway mediated by the RNA helicases MDA5 (IFIH1, interferon-induced helicase C domain containing protein 1) and RIG1 (Retinoic acid inducible gene 1) are over-expressed. Also over-expressed are MDA5 and LGP2 (a likely ortholog of mouse D11lgp2), gene that is known to be a negative regulator of the RIG1 pathway [110]. PKR, a double stranded RNA activated protein kinase, is also increased in expression. In the TLR3-signaling pathway, TICAM1, the adaptor molecule for TLR3-[111] is increased in expression. These four pathways share a common characteristic in that the natural agonists of these pathways are RNA-based, single stranded RNA (ssRNA) for TLR7 [11]; double stranded RNA (dsRNA) for TLR3 [112], PKR [113] and MDA5; and ssRNA and dsRNA for RIG1 [114]. Thus, the data indicate that signaling through TLR7 may potentially prime and amplify signaling through TLR3, PKR, and the cytosolic antiviral pathway. The net effect of this may be an optimization of the anti-viral response against RNA viruses. This conclusion is consistent with the synergy between various TLRs including TLR7 and TLR3 observed for the induction of cytokines and cytotoxic T cell responses [115,116].

## Conclusion

In this study, we have made the first comprehensive global gene expression analysis of high purity pDC stimulated with TLR7 agonists. Even though the induction of type 1 interferons by TLR7 agonists in pDC has been well documented, we show the first evidence that a variety of IFNA subtypes as well as IFNB genes are induced. Given the evidence that IFNA subtypes differ in potency in their anti-viral and anti-tumor activities, the induction of a variety of IFNA subtypes and IFNB by TLR7 agonists may be an advantage over therapies that utilize a single IFNA subtype. In addition to increased expression of type 1 interferons, which can prime other immune and non-immune cells for anti-viral response, pDC produce a large number of chemokines and cytokines which can recruit and modulate the activity of a variety of cells of the innate and adaptive immune systems. The data also provides evidence for autocrine transcriptional regulation which results in increased expression of a large number of interferon-inducible genes with known anti-viral activity, thus indicating a strong antiviral role of this cell type. Potential autocrine regulation by chemokines is also implicated in the large number of genes regulated by other transcription factors such as CREB1. In addition, the increased expression of the interferon-inducible gene ISG15, and several enzymes responsible for ISGlyation as well as genes know to be ISGylated, underscores the importance of the ISGylation pathway in activated pDC. The exact role(s) protein ISGylation plays in activated pDC is not clear. The fact that several substrates of ISG15 which are also known to have anti-viral activity are increased in expression suggests a potential role for protein ISGylation in the anti-viral activity of pDC. Even as early as 4 hr post stimulation, the gene expression data provides evidence for increased capacity for antigen processing and presentation and T cell co-stimulatory capacity. A distinct anti-apoptotic gene expression finger print also shows that stimulation with TLR7 agonists programs pDC for increased cell survival.

Finally, the data demonstrate the capacity for TLR7-mediated amplification of the innate immune response in pDC. Stimulation of the TLR7 pathway results in increased expression of components of TLR7-signaling. In addition, signaling components of three other innate anti-viral pathways which respond to RNA viruses, TLR3, PKR and the cytosolic RIG1/MDA5 pathway are also increased in expression suggesting priming to maximize anti-viral response towards RNA viruses. While the data demonstrates the capacity of the innate immune response to amplify itself, increased expression of negative regulators of the three pathways indicates a tight regulation so as to prevent uncontrolled inflammation.

In summary, the gene expression analysis in this study provides valuable insight into the molecular pathways and processes altered in pDC upon stimulation with TLR7 agonists. The study also underscores the powerful immune stimulatory capacity of synthetic TLR7 agonists and their potential and demonstrated use in anti-viral and anti-tumor therapies. 3M-852A is currently in phase II clinical study for cancer therapy.

## Methods

### TLR Agonists

Small molecule synthetic TLR7 agonist (3M-852A, N- [4-(4-amino-2-ethyl-1H-imidazo[4,5-c]quinolin-1-yl)butyl-]methanesulfonamide; formula, C_17_H_23_N_5_O_2_S; m.w., 361; and TLR7/8 agonist 3M-011, N-{2- [4-amino-2-(ethoxymethyl)-1H-imidazo[4,5-c]quinolin-1-yl]-1,1-imethylethyl}methanesulfonamide; formula, C_18_H_25_N_5_O_3_S, m.w., 391.5 were prepared by 3M Pharmaceuticals. Compounds were dissolved in dimethyl sulfoxide (DMSO, Sigma-Aldrich, St Louis, MO, USA) at a concentration of 10 mM for use in stimulation of cells.

### Preparation of Plasmacytoid Dendritic Cells (pDC)

Human peripheral blood mononuclear cells (PBMC) were obtained from healthy volunteers using an aphaeresis procedure at the Memorial Blood Center, Minneapolis MN. Subjects provided informed consent under an institutional review board prior to blood donation. PBMC were prepared by Ficoll gradient (Ficoll Paque™, Amersham Bioscience, Uppsala Sweden) as described previously [14]. Purification of pDC was done by positive selection using Miltenyi BDCA4+ Cell selection Kits, (Miltenyi Biotec, Auburn, CA), according to manufacturer's recommendations. BDCA-4 magnetic beads kit reagents were added at the recommended ratios, and incubated for 15 minutes at 10°C. Labeled cells were washed, and then passed through a D type column in a SuperMAX magnet. After extensive washing, enriched cells were centrifuged and labeled for flow sorting using, allophycocyanin (APC) labeled anti-CD123, FITC-labeled anti-BDCA2 antibodies (Miltenyi Biotec, Auburn, CA), and phycoerythrene (PE) labeled anti-CD11c, (Becton Dickinson Biosciences, Franklin Lakes, NJ). Cells were labeled for 20 minutes at 20°C, washed in 15 volumes of Hank's Balanced Salt Solution (HBSS), 25mM HEPES (Biosource, state, country), 2% Human Serum (Cambrex Bio Science, Walkersville MD). Flow sorting was performed on a FACSAria™ flow cytometer (Becton Dickinson Co., Franklin Lakes, NJ). Cells were collected into Iscove's Modified Dulbecco's Medium (IMDM), with 10% human serum (Cambrex Bio Science, East Rutherford, New Jersey) and allowed to rest for two hours prior to stimulation. Flow purified cells used for stimulation with TLR agonists were 99% or greater in purity as determined by the percentage of CD123+/BDCA2+/CD11c- cells. pDC were stimulated with 5 μM each of 3M-852A or 3M-011 dissolved in DMSO or the same volume of DMSO. The final concentration of DMSO was 0.1%. After four hours of stimulation, cells were harvested by centrifugation and RNA extracted as outlined below. 3M-852A and 3M-011 were evaluated in pDC obtained from the same two donors. In addition, 3M-852A was evaluated in pDC from two other donors.

### Analysis of Secreted Cytokines and Chemokines

Cell culture supernatants collected 4 hr after stimulation of pDC were frozen and later evaluated for TNFα, IFNα, and IL-12 protein. Human IFNα was measured by ELISA, (PBL Biomedical Laboratories, Piscataway, NJ). TNFα and IL-12 were measured by ORIGEN (IGEN) assay (BioVeris Corp, Gaithersburg, MD) [14]. In addition, supernatants from two experiments were evaluated using a Human Cytokine 25-Plex Luminex™ kit (Biosource, Camarillo, CA). Cytokines assayed were; IL-1β, IL-1ra, IL-2, IL-2R, IL-4, IL-5, IL-6, IL-7, IL-8, IL-10, IL-12p40/p70, IL-13, IL-15, IL-17, TNFα, IFNα, IFNγ, GM-CSF, MIP-1α, MIP-1β, IP-10, MIG, Eotaxin, RANTES, and MCP-1.

### RNA extraction and Reverse Transcription

RNA was extracted from cell pellets using the Qiagen RNeasy kit (Qiagen Inc., Valencia CA), according to manufacturer's recommendations. Purified RNA was reverse transcribed using SuperScript Double Stranded cDNA Synthesis kit (Invitrogen Corp, Gaithersburg MA) using a GeneChip^® ^T7-Oligo(dT) Promoter Primer Kit (Affymetrix Inc. Santa Clara CA) according to manufacturer's recommendations.

### Quantitative Polymerase Chain Reaction

Real time Reverse Transcriptase Polymerase Chain Reaction (RT-PCR) was performed using custom TaqMan^® ^Low Density Arrays (Applied Biosystems, Foster City, CA). Each Low Density Array contained TaqMan^® ^reagents for 23 different genes and GAPDH. Primers and probes for IFN subtypes IFNA2, IFNA8, IFNA1/13, IFNA14, IFNA21, and IFNB1 were purchased as pre-developed reagents from Applied Biosystems. Primers and probes for IFN subtypes IFNA5, IFNA7 and IFNA10 were designed in house using Primer Express Software (Applied Biosystems, Foster City CA) and custom made by Applied Biosystems. The following primer/probe sets were used: IFNA5; forward primer -TGTGATCTGCCTCAGACC-, reverse primer-GATTCTTCCCATTTGTGCCATTA-, TaqMan probe AACAGGAGGACTTTGATG; IFNA7, forward primer -GGTAGCCTAGTGATATTTG-, reverse primer -GATGGATTTGTAGCTGAGT-, TaqMan probe -CCTTTTCTTTACTGATG-; IFNA10, forward primer -GTTATCCATCTCAAGTAGCCT-, reverse primer -GATTTGTAGCTGAGCACCA-, TaqMan probe -TCTTTACTTATGGCCGTG-. TaqMan^® ^probes were FAM and MGB labeled. PCR was performed for thirty-five cycles of 30sec at 95°C and 1 min at 60°C, preceded by a 2 min at 50°C and 10 min. incubation at 95°C, using an ABI PRIZM 7900HT Sequence Detection System (Applied Biosystems, Foster City CA) and analyzed according to manufacturer's instructions. Relative quantitation of gene expression was performed according to the ΔΔCt method using GAPDH for normalization (User Bulletin #2, PE Applied Systems 1]. Fold change in expression due to stimulation with TLR agonists was calculated relative to vehicle- stimulated samples.

### Affymetrix GeneChip Analysis

Samples for Affymetrix GeneChip analysis were prepared by one round of amplification according to manufacturer's instructions. The cDNAs were amplified and biotin labeled using GeneChip^® ^Expression 3'-Amplification Reagents for IVT Labeling (Affymetrix Inc. Santa Clara, CA). Hybridization onto Affymetrix U133A Human GeneChip^® ^Arrays (Affymetrix Inc, Santa Clara, CA) washing and image acquisition was performed according to the manufacturer's recommendations. Image analysis was performed using Gene Chip Operating Software (GCOS). (Affymetrix Inc. Santa Clara, CA). The quality of the images was ascertained by monitoring the noise, background, percent transcript present, and the 3'/5' ratio for the housekeeping gene glyceraldehyde-3-phosphate dehydrogenase (GAPDH). 3'/5' ratios for GAPDH were 0.9 ± 0.2 and were within acceptable value of 1. GeneChips from each donor were normalized to their respective vehicle control chips for determination of fold change in expression. The data were imported into Data Mining Tools, (DMT) and filtered based on a signal detection p-value < 0.01, and signal log ratio (log 2 fold change) < -1.5 or > 1.5. Probes for which one or more of the samples passed these criteria were brought into Microsoft^® ^Excel where the list was further filtered. Probes that did not show consistent signal detection p-value (less than 0.01) and an expression change p-value (less than 0.005) across replicates for each TLR agonist was eliminated. Redundant Affymetrix probes were manually removed from the data leaving 680 unique genes that showed consistent change across replicates for at least one of the TLR agonists.

Cluster analysis of the 680 genes was performed with Spotfire DecisionSite-8.1 for Functional Genomics (Spotfire Inc, Somerville, MA), using the Unweighted Pair-Group Method with Arithmetic mean (UPGMA) and the Euclidean similarity measure. Several tools were used to understand the gene functions and the biological processes represented in the data. Gene Ontology classification was made using the Ontology Browser in Spotfire Decisions Site for Genomcis, as well as the network interactions software MetaCore (described below). Gene Ontology files were downloaded from the gene ontology website [117] for use in Spotfire. The ontology browser calculates a Fisher's Exact Test p-value, which reflects the chance that the gene ontology category is represented by random chance [118]. P-value < 0.05 is considered significant.

### Functional analysis of expression data

Gene regulatory networks were generated using MetaCore analytical suite version 4.2 build 8168 (GeneGo, St Joseph, MI). MetaCore is a web-based suite for functional analysis of experimental data in the context of manually curated human protein interactions, canonical pathways, and knowledge base ontologies of cellular processes, diseases, and toxicology. The database includes over 160,000 human protein interactions and metabolic reactions, as well as known drugs, metabolites, nutrients and other bioactive compounds. The experimental data in MetaCore can be subjected to enrichment analysis [119] in 6 functional ontologies including GeneOntology processes (GO), GeneGo process networks, Diseases, GeneGo Diseases, Canonical pathway maps and Metabolic processes. Enrichment analysis in GO processes was used in this study. Both enrichment analysis and calculation of statistical significance of networks are based on p-values which are defined as the probability of a given number of genes from the input list to match a certain number of genes in the ontology's folder, or the probability of the network's assembly from a random set of nodes (genes) the same size as the input list (described in the supplimentary files in [120] . The whole data set of 680 genes was used to build networks using the Analyze networks (transcription regulation) algorithm and Analyze networks (receptor) networks algorithm which generate sub-networks centered on transcription factors and receptors respectively. The sub-networks were scored and prioritized based on relative enrichment with the data from input list and saturation with "canonicl pathways" using p-values and z-scores as statistical metrics [120]. Networks of interest thus obtained were further built by merging different networks and or expanding interactions around a given object (protein/gene). Some networks were built using the shortest path (SP) algorithm (directed graph) using selected smaller data sets of 3 to 40 genes.

## Abbreviations

pDC–plasmacytoid dendritic cells, TLR–Toll like receptors, IFN–interferon, RT-PCR–Reverse Transcriptase Polymerase Chain Reaction,

## Competing interests

None of the authors have competing interests. The study reported in this manuscript was funded by 3M Pharmaceuticals, St Paul, MN. Barbara J. Bulbulian, Christie L. Riter and Dr. John P. Vasilakos were employees of 3M Pharmaceuticals and have left the company since the completion of the study. Raymond M. Gleason, Dr. Kenneth E. Lipson, and Dr. Woubalem Birmachu are current employees of 3M Company. Dr. Yuri Nikolsky, is an employee of GeneGo.

## Authors' contributions

RMG, BJB, and CLR contributed to sample preparation and flow analysis. RMG performed Affymetrix data acquisition and preliminary data analysis. Project conception, data mining, network analysis, and writing of the manuscript were performed by WB. JPV, KEL and YN contributed intellectual content and to revision of the manuscript. All authors approved the manuscript.

## Supplementary Material

Additional file 1 Genes altered in expression 4 hr post-stimulation of pDC with 3M-852A and 3M-011. This file summarizes the log2 fold change in expression for 680 genes altered in expression upon treatment of pDC with 3M-852A and 3M-011, 4 hr post stimulation. Fold change in expression was calculated with respect to vehicle-treated samples from the same donor. Genes were selected if the probes sets had signal detection p-value less than 0.01 and an expression change p-value less than 0.005 across replicates for each TLR agonist. Click here for file

Additional file 2 Legend describing symbols used in networks. Legend provides a key to the symbols used in the MetaCore networks. Click here for file
