# Supplementary material for: Transcriptional networks in plasmacytoid dendritic cells stimulated with synthetic TLR 7 agonists
Source: BMC Immunol. 2007 Oct 12;8:26. doi: 10.1186/1471-2172-8-26 (PMC2175514; doi:10.1186/1471-2172-8-26)
Supplement: Additional file 1 — Genes altered in expression 4 hr post-stimulation of pDC with 3M-852A and 3M-011. This file summarizes the log2 fold change in expression for 680 genes altered in expression upon treatment of pDC with 3M-852A and 3M-011, 4 hr post stimulation. Fold change in expression was calculated with respect to vehicle-treated samples from the same donor. Genes were selected if the probes sets had signal detection p-value less than 0.01 and an expression change p-value less than 0.005 across replicates for each TLR agonist. [file 1471-2172-8-26-S1.doc]

Additional File 1. Genes altered in expression 4 hr post-stimulation of pDC with 3M-852A and 3M-011

| **Affymetrix ID** | **Gene Symbol** | **3M-852A** | | | | **3M-011** | | **Gene Title** |
| --- | --- | --- | --- | --- | --- | --- | --- | --- |
|  |  | **D1** | **D2** | **D3** | **D4** | **D1** | **D2** |  |
| 216575_at | --- | 5.1 | 8.8 | 5.6 | 6.2 | 4.3 | 8.2 | --- |
| 210230_at | --- | -1.0 | 1.5 | -0.4 | -0.4 | -1.5 | 0.9 | Hypothetical LOC388388 |
| 214349_at | --- | -1.7 | 0.9 | -1.2 | -0.9 | -2.0 | 0.0 | Hypothetical LOC388388 |
| 221844_x_at | --- | -1.4 | -0.9 | -1.2 | -1.2 | -1.3 | -1.0 | CDNA clone IMAGE:6208446 |
| 212829_at | --- | -1.8 | -1.8 | -1.9 | -1.4 | -0.9 | -0.6 | CDNA FLJ13267 fis, clone OVARC1000964 |
| 213294_at | --- | 1.5 | 0.9 | 2.5 | 2.9 | 1.6 | 1.1 | Hypothetical protein FLJ38348 |
| AFFX-M27830_M_at | --- | -2.1 | -0.6 | -0.9 | -1.8 | -2.1 | -1.0 | LOC440118 |
| AFFX-M27830_5_at | --- | -2.4 | 0.7 | -2.1 | -0.7 | -1.9 | 0.6 | SRY (sex determining region Y)-box 18 |
| 212387_at | --- | -2.6 | -2.2 | -1.7 | -2.3 | -2.0 | -1.8 | Transcription factor 4 |
| 217757_at | A2M | 1.3 | 2.8 | 1.0 | 1.1 | 1.8 | 4.0 | alpha-2-macroglobulin |
| 209459_s_at | ABAT | 3.0 | 1.7 | 1.8 | 2.9 | 1.6 | 1.1 | 4-aminobutyrate aminotransferase |
| 213497_at | ABTB2 | 2.4 | 3.3 | 2.9 | 2.6 | 2.3 | 2.7 | ankyrin repeat and BTB (POZ) domain containing 2 |
| 201963_at | ACSL1 | 1.7 | 2.4 | 2.3 | 1.4 | 0.7 | 1.4 | acyl-CoA synthetase long-chain family member 1 |
| 201660_at | ACSL3 | -1.9 | -1.3 | -0.9 | -1.6 | -1.1 | -0.8 | Acyl-CoA synthetase long-chain family member 3 |
| 213214_x_at | ACTG1 | -1.2 | -1.6 | -2.2 | -1.5 | -1.1 | -0.5 | actin, gamma 1 |
| 202381_at | ADAM9 | 1.5 | 1.7 | 1.9 | 2.1 | 1.4 | 0.0 | ADAM metallopeptidase domain 9 (meltrin gamma) |
| 201786_s_at | ADAR | 1.3 | 1.0 | 1.9 | 1.7 | 1.3 | 1.1 | adenosine deaminase, RNA-specific |
| 203741_s_at | ADCY7 | -1.0 | -2.0 | -2.2 | -2.4 | -1.3 | -1.4 | adenylate cyclase 7 |
| 201034_at | ADD3 | -1.3 | -1.5 | -2.1 | -1.3 | -1.1 | 0.0 | adducin 3 (gamma) |
| 200903_s_at | AHCY | -1.7 | -1.4 | -0.7 | -1.3 | -1.6 | -1.5 | S-adenosylhomocysteine hydrolase |
| 202820_at | AHR | 1.1 | 2.1 | 1.8 | 1.9 | 0.6 | 1.4 | aryl hydrocarbon receptor |
| 210962_s_at | AKAP9 | -1.0 | -2.1 | -2.0 | -1.8 | -0.5 | -1.4 | A kinase (PRKA) anchor protein (yotiao) 9 |
| 201951_at | ALCAM | 1.7 | 2.2 | 1.2 | 2.3 | 1.1 | 1.5 | activated leukocyte cell adhesion molecule |
| 201425_at | ALDH2 | -1.5 | -1.8 | -1.5 | -1.8 | -1.4 | -1.4 | aldehyde dehydrogenase 2 family (mitochondrial) |
| 201196_s_at | AMD1 | 2.2 | 1.7 | 1.3 | 2.0 | 1.4 | 1.3 | adenosylmethionine decarboxylase 1 |
| 222108_at | AMIGO2 | 2.4 | 1.9 | 1.1 | 1.1 | 1.8 | 2.4 | adhesion molecule with Ig-like domain 2 |
| 221825_at | ANGEL2 | -1.4 | -1.5 | -0.8 | -1.4 | -1.4 | -1.1 | angel homolog 2 (Drosophila) |
| 218093_s_at | ANKRD10 | -1.2 | -2.0 | -1.2 | -1.3 | -1.1 | -1.9 | ankyrin repeat domain 10 |
| 216563_at | ANKRD12 | -1.6 | -1.7 | -2.3 | -1.8 | 0.0 | -0.7 | Ankyrin repeat domain 12 |
| 201012_at | ANXA1 | -1.1 | -0.9 | -2.1 | -1.6 | 0.0 | 0.0 | annexin A1 |
| 201302_at | ANXA4 | -2.1 | -2.4 | -1.4 | -2.2 | 0.0 | 0.0 | annexin A4 |
| 203350_at | AP1G1 | 1.9 | 1.2 | 1.4 | 1.4 | 1.0 | 1.1 | adaptor-related protein complex 1, gamma 1 subunit |
| 202120_x_at | AP2S1 | -1.6 | -0.7 | -0.3 | -1.1 | -1.0 | -0.7 | adaptor-related protein complex 2, sigma 1 subunit |
| 200011_s_at | ARF3 | -1.2 | -1.7 | -1.8 | -1.8 | -1.0 | -1.4 | ADP-ribosylation factor 3 |
| 209435_s_at | ARHGEF2 | 1.6 | 1.6 | 1.5 | 1.7 | 1.6 | 1.3 | rho/rac guanine nucleotide exchange factor (GEF) 2 |
| 218501_at | ARHGEF3 | 1.8 | 1.8 | 2.0 | 2.5 | 3.1 | 2.7 | Rho guanine nucleotide exchange factor (GEF) 3 |
| 202548_s_at | ARHGEF7 | -0.8 | -1.4 | -1.0 | -0.9 | -0.9 | -1.2 | Rho guanine nucleotide exchange factor (GEF) 7 |
| 201881_s_at | ARIH1 | 1.7 | 1.0 | 1.0 | 1.1 | 1.5 | 0.8 | ariadne homolog, ubiquitin-conjugating enzyme E2 binding protein, 1 (Drosophila) |
| 202641_at | ARL3 | -1.6 | -2.2 | -1.1 | -1.6 | -0.8 | -1.5 | ADP-ribosylation factor-like 3 |
| 202207_at | ARL4C | -4.2 | -3.8 | -2.7 | -3.3 | -3.5 | -3.5 | ADP-ribosylation factor-like 7 |
| 201954_at | ARPC1B /// LOC653888 | -1.7 | -1.4 | -1.5 | -1.2 | -1.1 | -0.8 | actin related protein 2/3 complex, subunit 1B, 41kDa |
| 214553_s_at | ARPP-19 | 1.6 | 0.8 | 1.0 | 1.2 | 1.6 | 0.8 | cyclic AMP phosphoprotein, 19 kD |
| 213702_x_at | ASAH1 | -2.5 | -2.1 | -2.4 | -2.3 | -2.4 | -1.5 | N-acylsphingosine amidohydrolase (acid ceramidase) 1 |
| 203428_s_at | ASF1A | 1.6 | 1.7 | 1.4 | 1.9 | 1.2 | 0.9 | ASF1 anti-silencing function 1 homolog A (S. cerevisiae) |
| 205047_s_at | ASNS | 1.7 | 1.4 | 1.5 | 0.6 | 1.3 | 0.0 | asparagine synthetase |
| 202672_s_at | ATF3 | 0.4 | 2.2 | 2.4 | 1.9 | 0.0 | 0.8 | activating transcription factor 3 |
| 201971_s_at | ATP6V1A | 2.1 | 0.9 | 0.5 | 2.3 | 2.1 | 0.8 | ATPase, H+ transporting, lysosomal 70kDa, V1 subunit A |
| 202874_s_at | ATP6V1C1 | 1.8 | 1.3 | 1.2 | 2.0 | 1.6 | 1.4 | ATPase, H+ transporting, lysosomal 42kDa, V1 subunit C, isoform 1 |
| 201772_at | AZIN1 | 1.6 | 1.0 | 0.5 | 1.5 | 1.1 | 0.8 | antizyme inhibitor 1 |
| 219326_s_at | B3GNT2 | 2.3 | 1.3 | 2.1 | 2.9 | 2.7 | 1.3 | UDP-GlcNAc:betaGal beta-1,3-N-acetylglucosaminyltransferase 1 |
| 221485_at | B4GALT5 | 1.4 | 1.4 | 1.7 | 1.5 | 1.2 | 1.4 | UDP-Gal:betaGlcNAc beta 1,4- galactosyltransferase, polypeptide 5 |
| 210818_s_at | BACH1 | 3.7 | 1.0 | 1.3 | 1.1 | 3.1 | 0.0 | BTB and CNC homology 1, basic leucine zipper transcription factor 1 |
| 202391_at | BASP1 | 2.4 | 2.1 | 1.4 | 2.3 | 2.2 | 2.3 | brain abundant, membrane attached signal protein 1 |
| 214452_at | BCAT1 | 2.7 | 3.7 | 1.7 | 3.6 | 2.5 | 3.7 | branched chain aminotransferase 1, cytosolic |
| 203685_at | BCL2 | 0.8 | 2.1 | 0.9 | 1.5 | 0.9 | 1.6 | B-cell CLL/lymphoma 2 |
| 205681_at | BCL2A1 | 3.1 | 5.5 | 4.5 | 5.5 | 2.7 | 5.0 | BCL2-related protein A1 |
| 206665_s_at | BCL2L1 | 8.0 | 5.2 | 6.0 | 5.9 | 7.6 | 4.9 | BCL2-like 1 |
| 208536_s_at | BCL2L11 | 3.0 | 3.4 | 1.9 | 4.6 | 2.3 | 1.3 | BCL2-like 11 (apoptosis facilitator) |
| 204908_s_at | BCL3 | -2.7 | -2.6 | -1.6 | -2.4 | -1.7 | 0.0 | B-cell CLL/lymphoma 3 |
| 201101_s_at | BCLAF1 | 1.7 | 0.9 | 0.7 | 1.2 | 1.3 | 0.8 | BCL2-associated transcription factor 1 |
| 202710_at | BET1 | 1.2 | 1.4 | 1.2 | 1.7 | 0.7 | 1.0 | BET1 homolog (S. cerevisiae) |
| 213154_s_at | BICD2 | -0.7 | -0.6 | -1.0 | -2.0 | -0.6 | -0.5 | bicaudal D homolog 2 (Drosophila) |
| 210538_s_at | BIRC3 | 2.5 | 2.8 | 2.6 | 2.9 | 2.0 | 2.4 | baculoviral IAP repeat-containing 3 |
| 206133_at | BIRC4BP | 2.7 | 1.6 | 3.4 | 4.8 | 2.9 | 2.0 | XIAP associated factor-1 |
| 208368_s_at | BRCA2 | 0.8 | 1.5 | 1.7 | 1.4 | 0.0 | 0.0 | breast cancer 2, early onset |
| 200920_s_at | BTG1 | -1.8 | -1.1 | -1.2 | -1.4 | -1.7 | -1.1 | B-cell translocation gene 1, anti-proliferative |
| 201236_s_at | BTG2 | -1.6 | -1.3 | -0.5 | -1.3 | -1.5 | -1.4 | BTG family, member 2 |
| 205548_s_at | BTG3 | 1.9 | 1.7 | 1.8 | 2.5 | 1.8 | 1.8 | BTG family, member 3 |
| 201457_x_at | BUB3 | 2.1 | 2.0 | 1.8 | 2.2 | 2.0 | 1.9 | BUB3 budding uninhibited by benzimidazoles 3 homolog (yeast) |
| 200776_s_at | BZW1 /// LOC151579 | 1.2 | 1.2 | 0.6 | 1.7 | 1.1 | 1.0 | basic leucine zipper and W2 domains 1 /// similar to basic leucine zipper and W2 domains 1 |
| 218789_s_at | C11orf71 | 1.3 | 1.4 | 1.8 | 1.4 | 0.0 | 0.0 | hypothetical protein FLJ20010 |
| 219806_s_at | C11orf75 | -2.7 | -2.8 | -1.6 | -2.2 | 0.0 | -2.2 | FN5 protein |
| 217448_s_at | C14orf92 | 2.7 | 1.6 | 0.7 | 2.0 | 2.3 | 1.6 | chromosome 14 open reading frame 92 /// similar to Epidermal Langerhans cell protein LCP1 |
| 204495_s_at | C15orf39 | -2.1 | -2.2 | -1.2 | -2.6 | -2.0 | -2.3 | DKFZP434H132 protein |
| 217967_s_at | C1orf24 | 2.2 | 3.0 | 2.1 | 2.3 | 1.5 | 2.4 | chromosome 1 open reading frame 24 |
| 220688_s_at | C1orf33 | 1.0 | 1.0 | 1.7 | 2.0 | 0.7 | 0.5 | chromosome 1 open reading frame 33 |
| 207571_x_at | C1orf38 | -2.4 | -2.5 | -2.9 | -3.2 | -2.3 | -2.3 | chromosome 1 open reading frame 38 |
| 209484_s_at | C1orf48 | -1.4 | -0.9 | -1.3 | -1.0 | -1.0 | -0.5 | chromosome 1 open reading frame 48 |
| 220199_s_at | C1orf80 | 1.2 | 0.6 | -0.7 | 0.9 | 2.1 | 1.4 | chromosome 1 open reading frame 80 |
| 221827_at | C20orf18 | 1.4 | 2.2 | 1.9 | 2.2 | 1.8 | 2.1 | chromosome 20 open reading frame 18 |
| 220941_s_at | C21orf91 | 0.8 | 1.2 | 1.5 | 1.9 | 0.0 | 1.4 | chromosome 21 open reading frame 91 |
| 209285_s_at | C3orf63 | -0.7 | -1.4 | -0.9 | -1.6 | -0.5 | -0.9 | retinoblastoma-associated protein 140 |
| 201310_s_at | C5orf13 | -2.4 | -2.5 | -3.7 | -3.8 | -1.6 | -1.8 | chromosome 5 open reading frame 13 |
| 222309_at | C6orf62 | 0.9 | 2.2 | 2.5 | 1.9 | 0.0 | 2.2 | Chromosome 6 open reading frame 62 |
| 219006_at | C6orf66 | 1.0 | 1.2 | 1.0 | 1.6 | 0.0 | 0.6 | chromosome 6 open reading frame 66 |
| 210815_s_at | CALCRL | 1.8 | 2.9 | 2.2 | 3.4 | 1.6 | 1.8 | calcitonin receptor-like |
| 214845_s_at | CALU | 2.1 | 0.6 | 0.4 | 1.6 | 2.2 | 0.0 | calumenin |
| 201850_at | CAPG | -2.0 | -1.1 | -1.2 | -1.7 | -1.4 | -0.7 | capping protein (actin filament), gelsolin-like |
| 213373_s_at | CASP8 | -1.9 | -2.3 | -2.6 | -2.3 | -2.1 | -1.8 | caspase 8, apoptosis-related cysteine peptidase |
| 212586_at | CAST | -1.4 | -2.0 | -2.4 | -2.3 | -0.9 | -1.0 | calpastatin |
| 201432_at | CAT | -1.9 | -1.5 | -2.1 | -1.9 | -1.2 | -1.1 | catalase |
| 209682_at | CBLB | -0.9 | -1.2 | -1.9 | -1.6 | -1.6 | -1.4 | Cas-Br-M (murine) ecotropic retroviral transforming sequence b |
| 200037_s_at | CBX3 /// LOC653972 | -1.1 | -1.2 | -1.2 | -1.0 | -0.9 | -0.5 | chromobox homolog 3 (HP1 gamma homolog, Drosophila) |
| 209479_at | CCDC28A | -1.5 | -1.4 | -1.7 | -1.8 | -1.0 | -0.7 | coiled-coil domain containing 28A |
| 218936_s_at | CCDC59 | 0.7 | 1.6 | 1.3 | 0.7 | 0.0 | 0.9 | HSPC128 protein |
| 205114_s_at | CCL3 /// CCL3L1 /// CCL3L3 /// LOC643930 | 2.5 | 2.8 | 5.9 | 5.9 | 0.8 | 1.6 | chemokine (C-C motif) ligand 3 /// chemokine (C-C motif) ligand 3-like 1 |
| 204103_at | CCL4 | 3.5 | 3.3 | 7.6 | 6.4 | 1.8 | 2.5 | chemokine (C-C motif) ligand 4 |
| 1405_i_at | CCL5 | 5.4 | 9.8 | 6.6 | 8.9 | 5.2 | 9.8 | chemokine (C-C motif) ligand 5 |
| 206337_at | CCR7 | 2.0 | 3.7 | 3.0 | 3.3 | 2.1 | 3.5 | chemokine (C-C motif) receptor 7 |
| 208653_s_at | CD164 | 1.9 | 1.5 | 1.0 | 2.6 | 1.6 | 1.5 | CD164 antigen, sialomucin |
| 204192_at | CD37 | -1.5 | -1.9 | -1.6 | -2.4 | -1.3 | -1.8 | CD37 antigen |
| 205692_s_at | CD38 | 2.2 | 2.0 | 2.8 | 3.6 | 3.0 | 2.6 | CD38 antigen (p45) |
| 205153_s_at | CD40 | 4.0 | 4.0 | 5.8 | 5.3 | 3.8 | 3.1 | CD40 antigen (TNF receptor superfamily member 5) |
| 204490_s_at | CD44 | 3.8 | 3.9 | 3.5 | 4.3 | 3.6 | 3.8 | CD44 antigen (homing function and Indian blood group system) |
| 208783_s_at | CD46 | -1.3 | -1.7 | -1.3 | -0.8 | -0.7 | -0.7 | membrane cofactor protein (CD46, trophoblast-lymphocyte cross-reactive antigen) |
| 213857_s_at | CD47 | -2.1 | -1.2 | -1.4 | -1.2 | -1.3 | -0.9 | CD47 antigen (Rh-related antigen, integrin-associated signal transducer) |
| 201925_s_at | CD55 | -1.0 | -1.0 | -0.7 | -1.7 | -1.3 | -1.1 | decay accelerating factor for complement (CD55, Cromer blood group system) |
| 211744_s_at | CD58 | 2.2 | 1.8 | 1.2 | 2.2 | 2.0 | 0.0 | CD58 antigen, (lymphocyte function-associated antigen 3) |
| 200983_x_at | CD59 | 1.2 | 1.1 | 0.6 | 1.7 | 1.2 | 1.2 | CD59 antigen p18-20 (antigen identified by monoclonal antibodies 16.3A5, EJ16, EJ30, EL32 and G344) |
| 200663_at | CD63 | -1.4 | -1.2 | -0.7 | -0.8 | -1.0 | -0.9 | CD63 antigen (melanoma 1 antigen) |
| 209795_at | CD69 | 1.5 | 4.6 | 2.8 | 5.1 | 1.6 | 4.1 | CD69 antigen (p60, early T-cell activation antigen) |
| 207176_s_at | CD80 | 2.0 | 3.0 | 2.4 | 2.7 | 1.3 | 2.4 | CD80 antigen (CD28 antigen ligand 1, B7-1 antigen) |
| 204440_at | CD83 | 0.4 | 1.4 | 2.8 | 2.1 | 0.5 | 1.3 | CD83 antigen (activated B lymphocytes, immunoglobulin superfamily) |
| 210895_s_at | CD86 | 3.2 | 5.0 | 3.4 | 4.2 | 2.7 | 4.1 | CD86 antigen (CD28 antigen ligand 2, B7-2 antigen) |
| 201005_at | CD9 | -2.0 | -0.5 | -1.5 | -1.1 | -1.6 | -0.4 | CD9 antigen (p24) |
| 201029_s_at | CD99 | -1.5 | -1.3 | -1.6 | -1.5 | -0.9 | -0.5 | CD99 antigen |
| 212899_at | CDC2L6 | -1.3 | -1.3 | -1.1 | -1.4 | -0.7 | -1.3 | cell division cycle 2-like 6 (CDK8-like) |
| 219343_at | CDC37L1 | 1.6 | 1.7 | 1.1 | 1.0 | 0.0 | 1.5 | CDC37 cell division cycle 37 homolog (S. cerevisiae)-like 1 |
| 218578_at | CDC73 | 1.3 | 1.2 | 1.3 | 0.9 | 0.0 | 1.0 | cell division cycle 73, Paf1/RNA polymerase II complex component, homolog (S. cerevisiae) |
| 201131_s_at | CDH1 | 0.5 | 1.5 | 1.4 | 1.4 | 0.0 | 0.8 | cadherin 1, type 1, E-cadherin (epithelial) |
| 201938_at | CDK2AP1 | -1.5 | -1.6 | -1.3 | -1.6 | -0.9 | -1.1 | CDK2-associated protein 1 |
| 213548_s_at | CDV3 | 2.0 | 0.9 | 0.5 | 1.2 | 1.7 | 0.6 | hypothetical protein H41 |
| 203098_at | CDYL | -0.6 | -1.2 | -1.8 | -1.3 | -1.5 | -1.3 | chromodomain protein, Y-like |
| 203973_s_at | CEBPD | 1.6 | 4.4 | 2.3 | 3.0 | 1.9 | 4.5 | CCAAT/enhancer binding protein (C/EBP), delta |
| 213618_at | CENTD1 | 1.4 | 2.8 | 2.0 | 1.6 | 1.3 | 1.8 | centaurin, delta 1 |
| 206003_at | CEP135 | 1.8 | 1.5 | 1.4 | 1.1 | 1.3 | 0.0 | centrosomal protein 4 |
| 210563_x_at | CFLAR | 2.5 | 3.2 | 2.5 | 3.7 | 2.6 | 3.1 | CASP8 and FADD-like apoptosis regulator |
| 219356_s_at | CHMP5 | 0.9 | 1.1 | 1.7 | 1.6 | 0.6 | 0.6 | chromatin modifying protein 5 |
| 206756_at | CHST7 | 2.9 | 5.4 | 3.5 | 3.5 | 2.5 | 4.6 | carbohydrate (N-acetylglucosamine 6-O) sulfotransferase 7 |
| 203044_at | CHSY1 | 1.0 | 0.6 | 0.9 | 1.4 | 1.4 | 1.7 | carbohydrate (chondroitin) synthase 1 |
| 201953_at | CIB1 | -2.1 | -1.5 | -1.3 | -1.6 | -1.6 | -0.9 | calcium and integrin binding 1 (calmyrin) |
| 200999_s_at | CKAP4 | 1.5 | 2.3 | 1.2 | 2.9 | 2.2 | 3.6 | cytoskeleton-associated protein 4 |
| 201897_s_at | CKS1B | -1.2 | -0.9 | -0.5 | -0.7 | -0.6 | -0.5 | CDC28 protein kinase regulatory subunit 1B |
| 220132_s_at | CLEC2D | 4.5 | 4.7 | 3.7 | 5.3 | 4.2 | 3.7 | C-type lectin domain family 2, member D |
| 217752_s_at | CNDP2 | -1.4 | -0.6 | -1.5 | -1.1 | -0.9 | 0.0 | CNDP dipeptidase 2 (metallopeptidase M20 family) |
| 218048_at | COMMD3 | -1.7 | -1.7 | -2.0 | -1.5 | -1.0 | -1.1 | COMM domain containing 3 |
| 208818_s_at | COMT | -1.8 | -1.6 | -1.2 | -1.3 | -1.1 | -0.7 | catechol-O-methyltransferase |
| 209083_at | CORO1A | -2.5 | -2.0 | -2.2 | -2.8 | -2.7 | -1.9 | coronin, actin binding protein, 1A |
| 202343_x_at | COX5B | -1.3 | -1.1 | -1.2 | -0.7 | -1.1 | -0.7 | cytochrome c oxidase subunit Vb |
| 201942_s_at | CPD | 3.2 | 2.1 | 2.0 | 2.7 | 2.8 | 1.6 | carboxypeptidase D |
| 202119_s_at | CPNE3 | -1.6 | -2.2 | -1.9 | -1.9 | -1.2 | -1.6 | copine III |
| 212345_s_at | CREB3L2 | -1.8 | -1.6 | -2.3 | -2.1 | -1.2 | -1.2 | cAMP responsive element binding protein 3-like 2 |
| 214508_x_at | CREM | 1.2 | 3.3 | 2.7 | 3.0 | 1.0 | 2.8 | cAMP responsive element modulator |
| 202950_at | CRYZ | -3.4 | -2.7 | -1.6 | -2.8 | -3.3 | -3.3 | crystallin, zeta (quinone reductase) |
| 205159_at | CSF2RB | -2.4 | -2.0 | -1.7 | -2.1 | -2.1 | -2.1 | colony stimulating factor 2 receptor, beta, low-affinity (granulocyte-macrophage) |
| 206075_s_at | CSNK2A1 | 1.8 | 0.9 | 0.3 | 1.2 | 1.7 | 0.8 | casein kinase 2, alpha 1 polypeptide |
| 207030_s_at | CSRP2 | 2.7 | 3.8 | 2.8 | 4.3 | 2.2 | 3.8 | cysteine and glycine-rich protein 2 |
| 203445_s_at | CTDSP2 | -1.9 | -2.0 | -1.9 | -2.2 | -1.4 | -1.6 | CTD (carboxy-terminal domain, RNA polymerase II, polypeptide A) small phosphatase 2 |
| 200764_s_at | CTNNA1 | 1.4 | 0.8 | 0.7 | 1.5 | 1.7 | 1.2 | catenin (cadherin-associated protein), alpha 1, 102kDa |
| 200838_at | CTSB | -1.1 | -1.4 | -1.6 | -0.7 | -0.7 | -1.0 | cathepsin B |
| 210042_s_at | CTSZ | -2.9 | -3.6 | -2.4 | -2.9 | -2.8 | -3.0 | cathepsin Z |
| 218097_s_at | CUEDC2 | -1.6 | -1.2 | -0.8 | -1.1 | -1.1 | -1.4 | CUE domain containing 2 |
| 204470_at | CXCL1 | 2.4 | 3.6 | 4.4 | 2.8 | 0.0 | 1.3 | chemokine (C-X-C motif) ligand 1 (melanoma growth stimulating activity, alpha) |
| 204533_at | CXCL10 | 3.7 | 6.7 | 6.4 | 7.9 | 4.3 | 7.1 | chemokine (C-X-C motif) ligand 10 |
| 211122_s_at | CXCL11 | 3.9 | 5.9 | 3.1 | 6.2 | 4.4 | 6.4 | chemokine (C-X-C motif) ligand 11 |
| 209774_x_at | CXCL2 | 0.6 | 1.3 | 4.8 | 2.6 | -1.3 | 0.0 | chemokine (C-X-C motif) ligand 2 |
| 207850_at | CXCL3 | 1.6 | 2.8 | 4.8 | 1.9 | -0.8 | 1.3 | chemokine (C-X-C motif) ligand 3 |
| 203915_at | CXCL9 | 2.7 | 3.7 | 2.1 | 3.4 | 2.4 | 3.2 | chemokine (C-X-C motif) ligand 9 |
| 217028_at | CXCR4 | -2.4 | -3.1 | -3.0 | -4.2 | -3.2 | -4.2 | chemokine (C-X-C motif) receptor 4 |
| 215785_s_at | CYFIP2 | -3.9 | -3.4 | -2.8 | -3.9 | -3.8 | -3.4 | cytoplasmic FMR1 interacting protein 2 |
| 213295_at | CYLD | -2.0 | -1.8 | -1.4 | -2.3 | -1.8 | -2.0 | Cylindromatosis (turban tumor syndrome) |
| 212527_at | D15Wsu75e | 2.3 | 2.7 | 1.6 | 2.5 | 1.8 | 2.2 | DNA segment, Chr 15, Wayne State University 75, expressed |
| 203139_at | DAPK1 | -1.6 | -2.1 | -1.8 | -1.5 | -1.4 | -2.0 | death-associated protein kinase 1 |
| 202428_x_at | DBI | -2.1 | -1.7 | -1.3 | -1.7 | -1.6 | -1.2 | diazepam binding inhibitor (GABA receptor modulator, acyl-Coenzyme A binding protein) |
| 203302_at | DCK | -1.3 | -1.2 | -1.1 | -0.9 | -0.7 | -0.7 | deoxycytidine kinase |
| 209231_s_at | DCTN5 | 1.3 | 0.9 | 0.6 | 1.9 | 1.2 | 0.6 | dynactin 5 (p25) |
| 214787_at | DENND4A | 2.6 | 2.2 | 2.5 | 1.9 | 1.8 | 1.2 | c-myc promoter binding protein |
| 205684_s_at | DENND4C | 2.3 | 1.0 | 0.9 | 1.0 | 2.3 | 1.5 | chromosome 9 open reading frame 55 |
| 210788_s_at | DHRS7 | -1.6 | -1.4 | -1.6 | -1.3 | -1.4 | -1.1 | dehydrogenase/reductase (SDR family) member 7 |
| 217989_at | DHRS8 | -2.7 | -1.4 | -2.3 | -1.8 | -1.7 | -1.0 | dehydrogenase/reductase (SDR family) member 8 |
| 211150_s_at | DLAT | 1.3 | 1.3 | 1.0 | 1.7 | 1.3 | 0.8 | dihydrolipoamide S-acetyltransferase (E2 component of pyruvate dehydrogenase complex) |
| 209095_at | DLD | 2.0 | 2.4 | 2.2 | 2.7 | 1.5 | 1.6 | dihydrolipoamide dehydrogenase (E3 component of pyruvate dehydrogenase complex, 2-oxo-glutarate complex, branched chain keto acid dehydrogenase complex) |
| 203811_s_at | DNAJB4 | 2.7 | 3.1 | 2.3 | 3.8 | 2.1 | 2.0 | DnaJ (Hsp40) homolog, subfamily B, member 4 |
| 204720_s_at | DNAJC6 | 3.0 | 2.9 | 2.5 | 2.3 | 2.6 | 2.6 | DnaJ (Hsp40) homolog, subfamily C, member 6 |
| 222154_s_at | DNAPTP6 | 1.8 | 1.5 | 2.0 | 2.9 | 2.7 | 2.5 | DNA polymerase-transactivated protein 6 |
| 205554_s_at | DNASE1L3 | -3.2 | -3.1 | -2.0 | -3.2 | -3.4 | -4.4 | deoxyribonuclease I-like 3 |
| 202776_at | DNTTIP2 | 1.2 | 1.5 | 1.8 | 1.3 | 0.0 | 0.8 | estrogen receptor binding protein |
| 205003_at | DOCK4 | 2.2 | 1.8 | 1.9 | 1.5 | 2.0 | 2.0 | dedicator of cytokinesis 4 |
| 200762_at | DPYSL2 | -1.9 | -1.5 | -1.6 | -1.9 | -1.5 | -0.5 | dihydropyrimidinase-like 2 |
| 201022_s_at | DSTN | -2.1 | -1.5 | -2.2 | -2.0 | -1.6 | -0.8 | destrin (actin depolymerizing factor) |
| 201536_at | DUSP3 | -1.3 | -0.9 | -1.2 | -0.9 | -1.0 | 0.0 | dual specificity phosphatase 3 (vaccinia virus phosphatase VH1-related) |
| 209932_s_at | DUT | -1.7 | -1.3 | -0.8 | -1.3 | -1.4 | -0.7 | dUTP pyrophosphatase |
| 201999_s_at | DYNLT1 | 1.3 | 2.5 | 1.9 | 2.2 | 1.6 | 2.8 | t-complex-associated-testis-expressed 1-like 1 |
| 219551_at | EAF2 | 2.4 | 3.1 | 1.8 | 2.5 | 1.7 | 2.7 | ELL associated factor 2 |
| 201750_s_at | ECE1 | 1.8 | 1.6 | 0.9 | 1.5 | 2.0 | 0.0 | endothelin converting enzyme 1 |
| 208091_s_at | ECOP | -2.1 | -1.6 | -1.3 | -1.7 | -1.4 | -1.3 | EGFR-coamplified and overexpressed protein /// EGFR-coamplified and overexpressed protein |
| 209572_s_at | EED | 1.0 | 1.7 | 1.4 | 1.7 | 0.8 | 1.4 | embryonic ectoderm development |
| 209536_s_at | EHD4 | 3.1 | 3.5 | 3.6 | 3.6 | 3.6 | 3.6 | EH-domain containing 4 |
| 204211_x_at | EIF2AK2 | 1.6 | 1.3 | 3.1 | 2.5 | 1.8 | 1.5 | eukaryotic translation initiation factor 2-alpha kinase 2 |
| 201144_s_at | EIF2S1 | 1.1 | 1.2 | 1.5 | 1.7 | 0.8 | 1.0 | eukaryotic translation initiation factor 2, subunit 1 alpha, 35kDa |
| 208708_x_at | EIF5 | 1.7 | 1.5 | 1.4 | 1.6 | 1.4 | 1.2 | eukaryotic translation initiation factor 5 |
| 209233_at | EMG1 | 0.4 | 1.1 | 0.6 | 1.7 | 0.0 | 1.1 | C2f protein |
| 203729_at | EMP3 | -1.1 | -0.8 | -0.4 | -0.4 | 0.0 | 0.0 | epithelial membrane protein 3 |
| 204160_s_at | ENPP4 | 3.0 | 2.4 | 2.9 | 1.8 | 2.1 | 1.7 | ectonucleotide pyrophosphatase/phosphodiesterase 4 (putative function) |
| 201216_at | ERP29 | -1.6 | -1.4 | -2.0 | -1.6 | -1.4 | -0.9 | endoplasmic reticulum protein 29 |
| 204774_at | EVI2A | 1.5 | 2.9 | 1.5 | 1.5 | 0.5 | 1.7 | ecotropic viral integration site 2A |
| 205061_s_at | EXOSC9 | 2.1 | 2.2 | 2.5 | 2.9 | 2.5 | 2.3 | exosome component 9 |
| 201995_at | EXT1 | 4.6 | 5.5 | 5.1 | 4.7 | 3.9 | 5.1 | exostoses (multiple) 1 |
| 221249_s_at | FAM117A | -2.3 | -1.6 | -1.7 | -2.4 | 0.0 | -1.6 | C/EBP-induced protein |
| 219629_at | FAM118A | -1.2 | -1.4 | -1.6 | -1.0 | 0.0 | -1.2 | chromosome 22 open reading frame 8 |
| 214946_x_at | FAM21B /// FAM21C /// RP11-56A21.1 /// LOC653450 | -0.6 | -1.0 | -1.7 | -0.9 | 0.0 | -0.7 | family with sequence similarity 21, member B/// similar to KIAA0592 protein |
| 208092_s_at | FAM49A | 1.8 | 2.3 | 1.6 | 2.4 | 1.6 | 1.7 | family with sequence similarity 49, member A |
| 220147_s_at | FAM60A | 1.5 | 1.7 | 1.3 | 1.2 | 1.6 | 1.7 | family with sequence similarity 60, member A |
| 219895_at | FAM70A | 2.1 | 2.6 | 2.1 | 2.7 | 1.3 | 2.4 | family with sequence similarity 70, member A |
| 203620_s_at | FCHSD2 | -2.5 | -1.7 | -1.2 | -2.3 | -1.9 | 0.0 | FCH and double SH3 domains 2 |
| 201798_s_at | FER1L3 | 3.9 | 4.6 | 2.1 | 4.2 | 3.6 | 4.9 | fer-1-like 3, myoferlin (C. elegans) |
| 203562_at | FEZ1 | 0.6 | 2.3 | 2.1 | 1.4 | 0.0 | 0.0 | fasciculation and elongation protein zeta 1 (zygin I) |
| 219117_s_at | FKBP11 | -0.6 | -1.9 | -0.7 | -0.8 | -0.9 | 0.0 | FK506 binding protein 11, 19 kDa |
| 218627_at | FLJ11259 | 1.5 | 2.0 | 1.9 | 2.3 | 1.6 | 1.1 | hypothetical protein FLJ11259 |
| 53720_at | FLJ11286 | 1.5 | 1.7 | 2.1 | 3.2 | 2.1 | 2.1 | hypothetical protein FLJ11286 |
| 218986_s_at | FLJ20035 | 2.4 | 2.5 | 4.7 | 4.3 | 2.7 | 2.7 | hypothetical protein FLJ20035 |
| 219258_at | FLJ20516 | 2.5 | 2.1 | 2.2 | 1.8 | 2.9 | 2.3 | timeless-interacting protein |
| 213940_s_at | FNBP1 | 2.4 | 2.7 | 2.2 | 2.4 | 2.1 | 2.5 | formin binding protein 1 |
| 215017_s_at | FNBP1L | 2.5 | 3.5 | 2.4 | 3.1 | 2.4 | 3.4 | formin binding protein 1-like |
| 204420_at | FOSL1 | 2.0 | 1.2 | 3.6 | 2.5 | 1.6 | 0.0 | FOS-like antigen 1 |
| 203091_at | FUBP1 | 1.6 | 1.0 | 0.6 | 1.3 | 1.7 | 1.0 | far upstream element (FUSE) binding protein 1 |
| 210178_x_at | FUSIP1 /// LOC642558 | 1.5 | 0.6 | 0.4 | 1.6 | 1.5 | 0.4 | FUS interacting protein (serine/arginine-rich) 1 |
| 200645_at | GABARAP | -1.6 | -1.5 | -1.4 | -1.7 | -1.3 | -1.3 | GABA(A) receptor-associated protein |
| 206173_x_at | GABPB2 | 2.0 | 1.9 | 1.4 | 2.5 | 1.7 | 1.2 | GA binding protein transcription factor, beta subunit 2 |
| 207574_s_at | GADD45B | 0.8 | 0.7 | 2.4 | 2.1 | 1.2 | 1.1 | growth arrest and DNA-damage-inducible, beta |
| 204417_at | GALC | -1.6 | -1.5 | -1.3 | -1.2 | 0.0 | -1.3 | galactosylceramidase (Krabbe disease) |
| 208693_s_at | GARS | 1.5 | 1.7 | 1.3 | 1.8 | 1.4 | 1.3 | glycyl-tRNA synthetase |
| 204224_s_at | GCH1 | 1.5 | 2.7 | 2.2 | 2.0 | 2.4 | 3.2 | GTP cyclohydrolase 1 (dopa-responsive dystonia) |
| 202923_s_at | GCLC | 2.1 | 3.1 | 2.2 | 2.2 | 1.2 | 2.5 | glutamate-cysteine ligase, catalytic subunit |
| 203925_at | GCLM | 1.8 | 2.4 | 1.6 | 2.8 | 1.3 | 2.0 | glutamate-cysteine ligase, modifier subunit |
| 204472_at | GEM | -2.4 | -1.4 | -0.2 | -2.7 | -2.5 | -1.8 | GTP binding protein overexpressed in skeletal muscle |
| 205527_s_at | GEMIN4 | 0.7 | 1.0 | 1.7 | 1.0 | 0.0 | 0.0 | gem (nuclear organelle) associated protein 4 |
| 214430_at | GLA | 1.9 | 2.9 | 2.3 | 3.4 | 1.8 | 2.7 | galactosidase, alpha |
| 207966_s_at | GLG1 | -1.6 | -1.8 | -2.2 | -1.9 | -1.6 | -1.8 | golgi apparatus protein 1 |
| 219015_s_at | GLT28D1 | 1.1 | 1.1 | 1.0 | 1.7 | 0.7 | 0.8 | glycosyltransferase 28 domain containing 1 |
| 202543_s_at | GMFB | 1.8 | 2.0 | 1.7 | 2.2 | 1.3 | 1.5 | glia maturation factor, beta |
| 204115_at | GNG11 | 3.1 | 2.8 | 3.4 | 2.6 | 2.6 | 2.4 | guanine nucleotide binding protein (G protein), gamma 11 |
| 211977_at | GPR107 | 1.0 | 1.6 | 1.2 | 1.2 | 0.7 | 1.2 | G protein-coupled receptor 107 |
| 204137_at | GPR137B | 0.4 | 1.7 | 1.6 | 2.0 | 0.0 | 1.2 | transmembrane 7 superfamily member 1 (upregulated in kidney) |
| 207651_at | GPR171 | 3.8 | 5.6 | 5.4 | 3.4 | 2.4 | 5.1 | G protein-coupled receptor 171 |
| 214467_at | GPR65 | -3.1 | -1.8 | -1.8 | -1.9 | -2.7 | -2.1 | G protein-coupled receptor 65 |
| 200736_s_at | GPX1 | -4.3 | -2.6 | -3.5 | -3.6 | -3.5 | -2.1 | glutathione peroxidase 1 |
| 200678_x_at | GRN | -1.1 | -1.1 | -1.1 | -1.7 | -0.9 | -1.0 | granulin |
| 205770_at | GSR | 2.1 | 2.0 | 1.4 | 2.4 | 2.2 | 1.6 | glutathione reductase |
| 202554_s_at | GSTM3 | 1.4 | 2.4 | 1.9 | 1.8 | 1.0 | 1.6 | glutathione S-transferase M3 (brain) |
| 201470_at | GSTO1 | -1.6 | -1.3 | -1.8 | -0.6 | -1.2 | -0.6 | glutathione S-transferase omega 1 |
| 206521_s_at | GTF2A1 | 1.7 | 1.3 | 1.3 | 1.1 | 1.7 | 0.0 | general transcription factor IIA, 1, 19/37kDa |
| 202354_s_at | GTF2F1 | 1.4 | 1.0 | 0.6 | 0.8 | 1.6 | 1.0 | general transcription factor IIF, polypeptide 1, 74kDa |
| 210891_s_at | GTF2I /// GTF2IP1 /// LOC649791 | -1.4 | -1.9 | -1.9 | -1.8 | -1.3 | -1.5 | general transcription factor II, i /// general transcription factor II, i, pseudogene 1 |
| 201338_x_at | GTF3A | -1.4 | -1.5 | -1.8 | -1.3 | -1.1 | -0.8 | general transcription factor IIIA |
| 218239_s_at | GTPBP4 | 1.1 | 1.4 | 1.7 | 1.7 | 0.5 | 0.7 | GTP binding protein 4 |
| 200075_s_at | GUK1 | -2.0 | -1.2 | -1.4 | -1.4 | -1.4 | -0.8 | guanylate kinase 1 /// guanylate kinase 1 |
| 211275_s_at | GYG1 | -1.4 | -0.9 | -1.1 | -0.9 | -1.2 | 0.0 | glycogenin |
| 207168_s_at | H2AFY | -1.5 | -1.3 | -1.3 | -1.3 | -1.3 | -1.5 | H2A histone family, member Y |
| 209273_s_at | HBLD2 | 0.9 | 1.5 | 0.8 | 0.9 | 1.4 | 1.8 | HESB like domain containing 2 |
| 201209_at | HDAC1 | -1.7 | -1.7 | -1.3 | -1.4 | -1.5 | -1.3 | histone deacetylase 1 |
| 200896_x_at | HDGF | -1.4 | -1.9 | -1.8 | -1.5 | -1.4 | -1.4 | hepatoma-derived growth factor (high-mobility group protein 1-like) |
| 203430_at | HEBP2 | -1.2 | -1.3 | -2.3 | -0.8 | 0.0 | 0.0 | heme binding protein 2 |
| 219863_at | HERC5 | 3.1 | 3.7 | 4.0 | 5.4 | 3.5 | 4.2 | hect domain and RLD 5 |
| 219352_at | HERC6 | 2.9 | 3.6 | 6.3 | 6.5 | 3.4 | 4.4 | hect domain and RLD 6 |
| 217168_s_at | HERPUD1 | -2.4 | -3.0 | -2.3 | -2.7 | -2.1 | -2.7 | homocysteine-inducible, endoplasmic reticulum stress-inducible, ubiquitin-like domain member 1 |
| 211267_at | HESX1 | 1.9 | 2.5 | 2.8 | 3.4 | 2.7 | 3.2 | homeo box (expressed in ES cells) 1 |
| 201944_at | HEXB | -1.5 | -1.0 | -1.3 | -0.6 | -1.1 | -0.6 | hexosaminidase B (beta polypeptide) |
| 210387_at | HIST1H2BG | -4.5 | -0.8 | -2.2 | -3.8 | 0.0 | -2.2 | histone 1, H2bg |
| 214290_s_at | HIST2H2AA /// LOC653610 /// H2A/R | 0.4 | 2.6 | 1.3 | 2.1 | 0.5 | 2.2 | histone 2, H2aa |
| 204512_at | HIVEP1 | -1.7 | -1.0 | -0.8 | -2.4 | -2.0 | -2.8 | human immunodeficiency virus type I enhancer binding protein 1 |
| 203932_at | HLA-DMB | -1.5 | -1.0 | -0.5 | -1.4 | -1.6 | -1.1 | major histocompatibility complex, class II, DM beta |
| 203290_at | HLA-DQA1 | 0.5 | 1.4 | 1.6 | 1.0 | 0.0 | 0.9 | major histocompatibility complex, class II, DQ alpha 1 |
| 214438_at | HLX1 | 1.5 | 1.3 | 2.4 | 1.1 | 1.6 | 1.7 | H2.0-like homeo box 1 (Drosophila) |
| 209377_s_at | HMGN3 | -1.9 | -1.5 | -1.9 | -1.2 | -1.4 | -1.6 | high mobility group nucleosomal binding domain 3 |
| 203665_at | HMOX1 | 0.6 | 1.6 | 1.9 | 2.3 | 0.5 | 1.2 | heme oxygenase (decycling) 1 |
| 206024_at | HPD | 1.0 | 2.2 | 2.0 | 2.6 | 0.0 | 0.0 | 4-hydroxyphenylpyruvate dioxygenase |
| 210112_at | HPS1 | 1.7 | 1.1 | 1.0 | 0.9 | 0.0 | 0.0 | Hermansky-Pudlak syndrome 1 |
| 205466_s_at | HS3ST1 | 2.2 | 7.0 | 2.7 | 4.0 | 2.1 | 6.4 | heparan sulfate (glucosamine) 3-O-sulfotransferase 1 |
| 201413_at | HSD17B4 | -1.6 | -2.2 | -1.6 | -2.0 | -1.7 | -1.8 | hydroxysteroid (17-beta) dehydrogenase 4 |
| 200800_s_at | HSPA1A /// HSPA1B | 0.6 | 1.2 | 1.4 | 1.7 | 0.9 | 0.9 | heat shock 70kDa protein 1A /// heat shock 70kDa protein 1B |
| 202581_at | HSPA1B | 0.4 | 1.2 | 1.3 | 1.2 | 0.0 | 1.2 | heat shock 70kDa protein 1B |
| 201841_s_at | HSPB1 | -1.7 | -1.6 | -0.9 | -0.8 | 0.0 | 0.0 | heat shock 27kDa protein 1 |
| 200042_at | HSPC117 | 0.9 | 1.0 | 1.0 | 1.5 | 1.2 | 1.6 | hypothetical protein HSPC117 |
| 200825_s_at | HYOU1 | -1.3 | -1.3 | -0.5 | -1.6 | -1.2 | -1.9 | hypoxia up-regulated 1 |
| 36564_at | IBRDC3 | 1.4 | 1.1 | 1.5 | 1.0 | 2.3 | 1.1 | IBR domain containing 3 |
| 201565_s_at | ID2 | 0.8 | 1.7 | 0.9 | 0.9 | 0.7 | 1.4 | inhibitor of DNA binding 2, dominant negative helix-loop-helix protein |
| 201566_x_at | ID2 /// ID2B | 1.9 | 3.5 | 3.7 | 2.6 | 1.1 | 2.8 | inhibitor of DNA binding 2, dominant negative helix-loop-helix protein |
| 212221_x_at | IDS | -1.9 | -1.6 | -2.2 | -3.0 | 0.0 | -2.3 | iduronate 2-sulfatase (Hunter syndrome) |
| 201631_s_at | IER3 | 0.7 | 0.8 | 3.1 | 2.6 | 0.7 | 1.1 | immediate early response 3 |
| 218611_at | IER5 | 0.4 | 1.6 | 1.3 | 1.4 | 0.0 | 1.4 | immediate early response 5 |
| 208966_x_at | IFI16 | 1.5 | 1.4 | 2.1 | 2.0 | 1.6 | 1.1 | interferon, gamma-inducible protein 16 |
| 202411_at | IFI27 | 1.7 | 5.1 | 2.5 | 4.5 | 1.9 | 4.4 | interferon, alpha-inducible protein 27 |
| 201422_at | IFI30 | -3.1 | -1.2 | -1.9 | -2.2 | -3.6 | -1.6 | interferon, gamma-inducible protein 30 |
| 209417_s_at | IFI35 | 1.3 | 1.7 | 2.6 | 3.2 | 2.2 | 2.2 | interferon-induced protein 35 |
| 214453_s_at | IFI44 | 2.2 | 2.5 | 4.4 | 4.8 | 2.5 | 2.3 | interferon-induced protein 44 |
| 204439_at | IFI44L | 2.6 | 2.2 | 5.0 | 5.4 | 2.5 | 2.0 | interferon-induced protein 44-like |
| 204415_at | IFI6 | 2.6 | 3.1 | 4.5 | 6.4 | 3.2 | 3.2 | interferon, alpha-inducible protein (clone IFI-6-16) |
| 219209_at | IFIH1 | 1.6 | 1.9 | 2.3 | 2.0 | 1.8 | 1.6 | interferon induced with helicase C domain 1 |
| 203153_at | IFIT1 | 4.4 | 5.1 | 7.3 | 9.6 | 5.2 | 6.0 | interferon-induced protein with tetratricopeptide repeats 1 |
| 217502_at | IFIT2 | 4.0 | 5.0 | 4.0 | 4.3 | 4.5 | 5.5 | interferon-induced protein with tetratricopeptide repeats 2 |
| 204747_at | IFIT3 | 2.8 | 3.9 | 3.8 | 5.4 | 3.6 | 4.4 | interferon-induced protein with tetratricopeptide repeats 3 |
| 203595_s_at | IFIT5 | 2.8 | 3.4 | 2.7 | 3.6 | 2.6 | 3.7 | interferon-induced protein with tetratricopeptide repeats 5 |
| 214022_s_at | IFITM1 | 2.2 | 3.7 | 3.8 | 5.2 | 2.7 | 3.8 | interferon induced transmembrane protein 1 (9-27) |
| 208375_at | IFNA1 | 6.0 | 10.6 | 7.1 | 7.3 | 4.6 | 11.2 | interferon, alpha 1 |
| 208261_x_at | IFNA10 | 5.3 | 8.1 | 4.6 | 5.5 | 4.1 | 7.6 | interferon, alpha 10 |
| 208344_x_at | IFNA13 | 5.4 | 7.8 | 2.9 | 5.6 | 4.0 | 7.5 | interferon, alpha 13 |
| 208182_x_at | IFNA14 | 6.0 | 9.5 | 4.4 | 7.2 | 4.2 | 8.9 | interferon, alpha 14 |
| 208448_x_at | IFNA16 | 6.1 | 5.9 | 5.2 | 7.2 | 4.3 | 5.5 | interferon, alpha 16 |
| 211405_x_at | IFNA17 | 5.0 | 5.4 | 4.1 | 5.2 | 3.4 | 4.9 | interferon, alpha 17 |
| 211338_at | IFNA2 | 6.2 | 7.2 | 6.4 | 11.5 | 4.3 | 6.7 | interferon, alpha 2 |
| 211145_x_at | IFNA21 | 6.6 | 8.3 | 5.5 | 8.5 | 5.1 | 7.4 | interferon, alpha 21 |
| 207964_x_at | IFNA4 | 5.6 | 6.2 | 4.3 | 6.9 | 4.1 | 5.7 | interferon, alpha 4 |
| 214569_at | IFNA5 | 5.9 | 6.6 | 5.5 | 7.8 | 4.5 | 6.5 | interferon, alpha 5 |
| 208259_x_at | IFNA7 | 5.7 | 6.1 | 4.2 | 6.9 | 4.1 | 5.5 | interferon, alpha 7 |
| 208173_at | IFNB1 | 6.9 | 8.2 | 8.2 | 7.8 | 4.8 | 6.9 | interferon, beta 1, fibroblast |
| 207817_at | IFNW1 | 4.7 | 6.4 | 4.8 | 5.1 | 2.7 | 5.9 | interferon, omega 1 |
| 201508_at | IGFBP4 | 4.6 | 4.3 | 5.5 | 4.6 | 5.0 | 4.8 | insulin-like growth factor binding protein 4 |
| 221651_x_at | IGKC /// IGKV1-5 /// LOC651928 | -1.6 | -0.8 | -0.5 | -1.3 | -1.5 | -1.9 | immunoglobulin kappa constant /// immunoglobulin kappa variable 1-5 |
| 204912_at | IL10RA | -2.3 | -2.3 | -1.1 | -1.7 | -2.2 | -2.5 | interleukin 10 receptor, alpha |
| 209575_at | IL10RB | -2.1 | -2.0 | -1.7 | -1.7 | -1.6 | -1.4 | interleukin 10 receptor, beta |
| 207072_at | IL18RAP | 0.4 | 1.3 | 2.2 | 1.9 | 0.0 | 0.0 | interleukin 18 receptor accessory protein |
| 220322_at | IL1F9 | 3.7 | 4.6 | 4.6 | 4.9 | 3.2 | 4.0 | interleukin 1 family, member 9 |
| 220054_at | IL23A | 3.8 | 4.3 | 4.2 | 4.9 | 2.2 | 3.8 | interleukin 23, alpha subunit p19 |
| 211269_s_at | IL2RA | 3.9 | 4.7 | 3.8 | 5.1 | 3.3 | 4.1 | interleukin 2 receptor, alpha |
| 205207_at | IL6 | 4.4 | 4.1 | 5.3 | 6.9 | 2.8 | 3.0 | interleukin 6 (interferon, beta 2) |
| 211506_s_at | IL8 | 2.9 | 2.6 | 3.7 | 6.0 | 1.8 | 1.9 | interleukin 8 |
| 210511_s_at | INHBA | 2.5 | 2.2 | 7.8 | 3.7 | 0.0 | 1.2 | inhibin, beta A (activin A, activin AB alpha polypeptide) |
| 201627_s_at | INSIG1 | 1.5 | 2.4 | 2.9 | 0.9 | 1.0 | 1.3 | insulin induced gene 1 |
| 218819_at | INTS6 | -1.6 | -0.7 | -0.5 | -0.7 | -1.3 | -0.9 | DEAD/H (Asp-Glu-Ala-Asp/His) box polypeptide 26 |
| 204057_at | IRF8 | -1.3 | -1.4 | -1.6 | -2.0 | -1.0 | -0.6 | interferon regulatory factor 8 |
| 205483_s_at | ISG15 | 2.0 | 3.6 | 5.6 | 5.0 | 2.5 | 3.8 | interferon, alpha-inducible protein (clone IFI-15K) |
| 204698_at | ISG20 | 2.3 | 3.8 | 7.5 | 5.3 | 2.8 | 4.1 | interferon stimulated exonuclease gene 20kDa |
| 203882_at | ISGF3G | 1.0 | 1.0 | 1.4 | 1.6 | 1.2 | 0.7 | interferon-stimulated transcription factor 3, gamma 48kDa |
| 205816_at | ITGB8 | 4.8 | 3.3 | 2.9 | 5.0 | 4.3 | 3.0 | integrin, beta 8 |
| 217731_s_at | ITM2B | -1.5 | -0.6 | -1.2 | -1.2 | 0.0 | 0.0 | integral membrane protein 2B |
| 221004_s_at | ITM2C | -2.1 | -1.3 | -2.0 | -1.6 | -1.5 | -0.9 | integral membrane protein 2C |
| 212492_s_at | JMJD2B | -1.9 | -1.1 | -1.0 | -1.6 | -1.4 | -0.8 | jumonji domain containing 2B |
| 203752_s_at | JUND | -1.3 | -1.2 | -1.3 | -1.8 | -1.0 | -1.0 | jun D proto-oncogene |
| 212188_at | KCTD12 | -2.8 | -0.6 | -1.9 | -2.8 | -3.9 | -1.5 | potassium channel tetramerisation domain containing 12 |
| 218474_s_at | KCTD5 | -1.3 | -1.7 | -1.4 | -1.6 | -1.3 | -1.5 | potassium channel tetramerisation domain containing 5 |
| 201729_s_at | KIAA0100 | -1.0 | -1.7 | -1.0 | -0.9 | -0.5 | -1.0 | KIAA0100 gene product |
| 202181_at | KIAA0247 | -2.0 | -2.2 | -1.5 | -1.9 | -1.9 | -1.6 | KIAA0247 |
| 212619_at | KIAA0286 | 2.5 | 1.8 | 0.7 | 3.7 | 1.8 | 0.8 | KIAA0286 protein |
| 212052_s_at | KIAA0676 | -1.3 | -1.2 | -1.7 | -0.8 | 0.0 | -0.8 | KIAA0676 protein |
| 204157_s_at | KIAA0999 | 0.9 | 1.4 | 1.4 | 1.1 | 0.5 | 1.0 | KIAA0999 protein |
| 209378_s_at | KIAA1128 | 2.6 | 2.2 | 1.7 | 2.2 | 2.6 | 2.5 | KIAA1128 |
| 213387_at | KIAA1240 | -1.0 | -1.7 | -1.2 | -1.2 | -0.5 | -1.0 | KIAA1240 protein |
| 203513_at | KIAA1840 | 2.5 | 2.3 | 2.4 | 2.1 | 1.7 | 1.6 | hypothetical protein FLJ21439 |
| 205306_x_at | KMO | 2.1 | 1.4 | 0.7 | 1.1 | 1.4 | 1.1 | kynurenine 3-monooxygenase (kynurenine 3-hydroxylase) |
| 213656_s_at | KNS2 | -1.9 | -1.5 | -1.3 | -1.6 | -2.0 | -1.0 | kinesin 2 |
| 202055_at | KPNA1 | 1.5 | 0.7 | 0.8 | 1.3 | 1.1 | 0.0 | Karyopherin alpha 1 (importin alpha 5) |
| 211762_s_at | KPNA2 /// LOC643995 | 1.5 | 1.5 | 1.1 | 1.7 | 0.8 | 1.0 | karyopherin alpha 2 (RAG cohort 1, importin alpha 1) /// karyopherin alpha 2 (RAG cohort 1, importin alpha 1) |
| 209653_at | KPNA4 | 2.3 | 1.2 | 1.0 | 2.1 | 2.0 | 0.7 | karyopherin alpha 4 (importin alpha 3) |
| 212102_s_at | KPNA6 | 1.7 | 0.8 | 0.8 | 0.8 | 1.7 | 0.3 | karyopherin alpha 6 (importin alpha 7) |
| 201553_s_at | LAMP1 | -1.4 | -1.0 | -0.6 | -1.0 | -1.2 | -0.9 | lysosomal-associated membrane protein 1 |
| 205569_at | LAMP3 | 2.8 | 3.0 | 4.6 | 5.0 | 3.0 | 3.7 | lysosomal-associated membrane protein 3 |
| 217933_s_at | LAP3 | 0.7 | 0.6 | 1.8 | 2.2 | 0.9 | 0.7 | leucine aminopeptidase 3 |
| 201721_s_at | LAPTM5 | -1.4 | -1.0 | -0.9 | -1.8 | -1.1 | -0.8 | lysosomal associated multispanning membrane protein 5 |
| 214155_s_at | LARP4 | 1.2 | 1.3 | 1.1 | 1.6 | 0.0 | 1.2 | La ribonucleoprotein domain family, member 4 |
| 202594_at | LEPROTL1 | -2.1 | -1.7 | -1.6 | -1.5 | -1.4 | -0.9 | leptin receptor overlapping transcript-like 1 |
| 208949_s_at | LGALS3 /// GALIG | 0.8 | 1.5 | 1.1 | 1.6 | 0.9 | 1.2 | lectin, galactoside-binding, soluble, 3 (galectin 3) /// galectin-3 internal gene |
| 219364_at | LGP2 | 1.5 | 2.7 | 4.8 | 2.7 | 2.5 | 3.1 | likely ortholog of mouse D11lgp2 |
| 215838_at | LILRA5 | 1.7 | 1.7 | 1.3 | 3.5 | 1.6 | 2.2 | leukocyte immunoglobulin-like receptor, subfamily A (with TM domain), member 5 |
| 200704_at | LITAF | 1.3 | 1.8 | 1.1 | 1.2 | 1.7 | 1.7 | lipopolysaccharide-induced TNF factor |
| 218191_s_at | LMBRD1 | -1.4 | -1.6 | -1.6 | -1.3 | -0.8 | -0.8 | LMBR1 domain containing 1 |
| 203276_at | LMNB1 | 2.1 | 2.4 | 1.4 | 1.3 | 2.7 | 3.3 | lamin B1 |
| 203622_s_at | LOC56902 | 1.7 | 1.3 | 1.9 | 2.3 | 0.9 | 0.8 | putatative 28 kDa protein |
| 222281_s_at | LOC642299 /// LOC651957 | -1.5 | -1.6 | -1.0 | -1.8 | -1.2 | -1.7 | --- |
| 202651_at | LPGAT1 | -1.4 | -1.2 | -1.7 | -1.9 | -1.4 | -1.0 | lysophosphatidylglycerol acyltransferase 1 |
| 212276_at | LPIN1 | -1.6 | -2.0 | -1.4 | -2.4 | 0.0 | -1.4 | lipin 1 |
| 220253_s_at | LRP12 | 1.9 | 1.4 | 1.9 | 5.2 | 1.2 | 0.0 | low density lipoprotein-related protein 12 |
| 208433_s_at | LRP8 | -1.1 | -1.9 | -0.9 | -1.4 | -1.1 | -1.7 | low density lipoprotein receptor-related protein 8, apolipoprotein e receptor |
| 201862_s_at | LRRFIP1 | -1.1 | -1.1 | -1.2 | -0.9 | -1.1 | -0.7 | leucine rich repeat (in FLII) interacting protein 1 |
| 202736_s_at | LSM4 | -2.2 | -1.9 | -1.7 | -1.0 | -1.3 | -1.4 | LSM4 homolog, U6 small nuclear RNA associated (S. cerevisiae) |
| 204559_s_at | LSM7 | -1.4 | -1.3 | -0.9 | -1.2 | -1.5 | -1.0 | LSM7 homolog, U6 small nuclear RNA associated (S. cerevisiae) |
| 206975_at | LTA | 7.0 | 4.7 | 7.6 | 6.2 | 5.3 | 3.5 | lymphotoxin alpha (TNF superfamily, member 1) |
| 208771_s_at | LTA4H | -2.0 | -2.5 | -2.3 | -2.0 | -2.0 | -1.7 | leukotriene A4 hydrolase |
| 207339_s_at | LTB | -3.2 | -2.5 | -1.4 | -1.8 | -3.0 | -3.1 | lymphotoxin beta (TNF superfamily, member 3) |
| 206584_at | LY96 | -3.2 | -1.1 | -1.9 | -2.0 | -3.0 | -1.3 | lymphocyte antigen 96 |
| 202625_at | LYN | 0.9 | 1.5 | 1.3 | 1.2 | 1.1 | 1.3 | v-yes-1 Yamaguchi sarcoma viral related oncogene homolog |
| 203518_at | LYST | 2.0 | 2.0 | 1.8 | 2.2 | 1.9 | 1.4 | lysosomal trafficking regulator |
| 218437_s_at | LZTFL1 | -1.4 | -1.8 | -2.0 | -2.8 | 0.0 | -1.6 | leucine zipper transcription factor-like 1 |
| 204970_s_at | MAFG | 1.4 | 1.9 | 1.5 | 1.8 | 0.9 | 1.2 | v-maf musculoaponeurotic fibrosarcoma oncogene homolog G (avian) |
| 208309_s_at | MALT1 | 1.2 | 2.3 | 0.6 | 2.1 | 0.8 | 1.6 | mucosa associated lymphoid tissue lymphoma translocation gene 1 |
| 203151_at | MAP1A | -1.2 | -1.4 | -0.9 | -2.0 | -1.2 | -1.4 | microtubule-associated protein 1A |
| 214786_at | MAP3K1 | 3.2 | 2.4 | 1.9 | 2.8 | 3.2 | 1.1 | mitogen-activated protein kinase kinase kinase 1 |
| 210284_s_at | MAP3K7IP2 | 2.1 | 0.8 | 1.0 | 1.7 | 1.8 | 0.8 | mitogen-activated protein kinase kinase kinase 7 interacting protein 2 |
| 205027_s_at | MAP3K8 | 2.9 | 3.4 | 3.8 | 3.1 | 1.6 | 2.4 | mitogen-activated protein kinase kinase kinase 8 |
| 206571_s_at | MAP4K4 | 1.4 | 2.1 | 2.0 | 1.4 | 0.8 | 1.8 | mitogen-activated protein kinase kinase kinase kinase 4 |
| 207121_s_at | MAPK6 | 1.6 | 1.8 | 1.8 | 1.7 | 1.2 | 1.5 | mitogen-activated protein kinase 6 |
| 201670_s_at | MARCKS | 3.0 | 4.4 | 4.0 | 3.8 | 2.4 | 3.9 | myristoylated alanine-rich protein kinase C substrate |
| 218440_at | MCCC1 | -1.5 | -2.1 | -1.4 | -1.9 | -2.2 | 0.0 | methylcrotonoyl-Coenzyme A carboxylase 1 (alpha) |
| 200798_x_at | MCL1 | 1.4 | 1.2 | 0.7 | 1.9 | 1.6 | 1.0 | myeloid cell leukemia sequence 1 (BCL2-related) |
| 204059_s_at | ME1 | 1.3 | 1.4 | 1.5 | 2.5 | 1.2 | 1.4 | malic enzyme 1, NADP(+)-dependent, cytosolic |
| 210154_at | ME2 | 1.5 | 0.3 | 0.7 | 1.1 | 1.7 | 0.8 | malic enzyme 2, NAD(+)-dependent, mitochondrial |
| 211801_x_at | MFN1 | 2.1 | 1.6 | 1.4 | 2.7 | 2.1 | 1.4 | mitofusin 1 |
| 218109_s_at | MFSD1 | -1.6 | -1.5 | -1.3 | -1.2 | -1.2 | -1.2 | major facilitator superfamily domain containing 1 |
| 201126_s_at | MGAT1 | -1.5 | -2.1 | -1.3 | -1.5 | -1.4 | -0.8 | mannosyl (alpha-1,3-)-glycoprotein beta-1,2-N-acetylglucosaminyltransferase |
| 214696_at | MGC14376 | 1.8 | 2.0 | 1.6 | 2.6 | 1.2 | 1.6 | hypothetical protein MGC14376 |
| 218376_s_at | MICAL1 | -1.4 | -1.1 | -1.6 | -1.8 | -1.5 | -0.9 | microtubule associated monoxygenase, calponin and LIM domain containing 1 |
| 218205_s_at | MKNK2 | -3.5 | -2.5 | -1.8 | -2.8 | -3.4 | -2.7 | MAP kinase interacting serine/threonine kinase 2 |
| 203414_at | MMD | 2.3 | 1.8 | 1.6 | 2.0 | 2.4 | 1.7 | monocyte to macrophage differentiation-associated |
| 204959_at | MNDA | 3.6 | 2.5 | 6.0 | 2.4 | 4.2 | 4.4 | myeloid cell nuclear differentiation antigen |
| 209928_s_at | MSC | 1.7 | 1.6 | 1.2 | 1.9 | 1.7 | 1.7 | musculin (activated B-cell factor-1) |
| 201761_at | MTHFD2 | 0.9 | 2.0 | 1.3 | 1.4 | 1.1 | 1.4 | methylenetetrahydrofolate dehydrogenase (NADP+ dependent) 2, methenyltetrahydrofolate cyclohydrolase |
| 220346_at | MTHFD2L | 1.8 | 2.2 | 2.9 | 1.0 | 0.0 | 0.8 | methylenetetrahydrofolate dehydrogenase (NADP+ dependent) 2-like |
| 202086_at | MX1 | 1.4 | 1.4 | 4.7 | 3.7 | 1.7 | 1.4 | myxovirus (influenza virus) resistance 1, interferon-inducible protein p78 (mouse) |
| 204994_at | MX2 | 1.3 | 1.9 | 2.3 | 2.9 | 1.6 | 2.0 | myxovirus (influenza virus) resistance 2 (mouse) |
| 204798_at | MYB | -1.8 | -1.4 | -1.8 | -2.3 | -1.5 | -1.5 | v-myb myeloblastosis viral oncogene homolog (avian) |
| 202431_s_at | MYC | 5.7 | 3.5 | 5.7 | 7.9 | 4.4 | 0.0 | v-myc myelocytomatosis viral oncogene homolog (avian) |
| 209124_at | MYD88 | 1.3 | 1.2 | 1.4 | 1.6 | 1.5 | 1.7 | myeloid differentiation primary response gene (88) |
| 204601_at | N4BP1 | 2.1 | 1.8 | 2.0 | 2.7 | 2.4 | 1.9 | Nedd4 binding protein 1 |
| 208919_s_at | NADK | -2.8 | -2.8 | -2.4 | -2.8 | -2.5 | -2.4 | NAD kinase |
| 201521_s_at | NCBP2 | 0.9 | 0.3 | 0.6 | 1.6 | 0.7 | 0.0 | nuclear cap binding protein subunit 2, 20kDa |
| 209949_at | NCF2 | 3.7 | 4.8 | 4.3 | 4.4 | 3.9 | 4.7 | neutrophil cytosolic factor 2 (65kDa, chronic granulomatous disease, autosomal 2) |
| 206790_s_at | NDUFB1 | -1.6 | -0.5 | -0.9 | -1.1 | -1.4 | -0.8 | NADH dehydrogenase (ubiquinone) 1 beta subcomplex, 1, 7kDa |
| 211752_s_at | NDUFS7 | -1.9 | -0.6 | -1.0 | -1.2 | -1.1 | -0.6 | NADH dehydrogenase (ubiquinone) Fe-S protein 7, 20kDa (NADH-coenzyme Q reductase) |
| 203189_s_at | NDUFS8 | -1.5 | -1.6 | -1.7 | -0.9 | -1.2 | -0.7 | NADH dehydrogenase (ubiquinone) Fe-S protein 8, 23kDa (NADH-coenzyme Q reductase) |
| 212445_s_at | NEDD4L | 1.1 | 1.1 | 1.7 | 1.4 | 1.1 | 1.6 | neural precursor cell expressed, developmentally down-regulated 4-like |
| 218888_s_at | NETO2 | 3.1 | 5.9 | 2.8 | 3.6 | 2.8 | 4.7 | neuropilin (NRP) and tolloid (TLL)-like 2 |
| 215092_s_at | NFAT5 | 2.4 | 2.2 | 2.3 | 1.8 | 2.0 | 2.0 | nuclear factor of activated T-cells 5, tonicity-responsive |
| 217526_at | NFATC2IP | -0.9 | -1.2 | -1.2 | -1.7 | -0.7 | -1.2 | nuclear factor of activated T-cells, cytoplasmic, calcineurin-dependent 2 interacting protein |
| 209239_at | NFKB1 | 2.5 | 2.6 | 2.9 | 3.1 | 2.4 | 2.4 | nuclear factor of kappa light polypeptide gene enhancer in B-cells 1 (p105) |
| 209636_at | NFKB2 | 1.3 | 0.4 | 1.8 | 0.6 | 1.5 | 0.0 | nuclear factor of kappa light polypeptide gene enhancer in B-cells 2 (p49/p100) |
| 204109_s_at | NFYA | 2.6 | 1.8 | 0.9 | 2.2 | 2.0 | 0.0 | nuclear transcription factor Y, alpha |
| 209075_s_at | NIFUN | -1.6 | -1.4 | -1.2 | -1.2 | -1.5 | -1.6 | NifU-like N-terminal domain containing |
| 203045_at | NINJ1 | 0.9 | 1.5 | 1.8 | 1.1 | 0.9 | 1.2 | ninjurin 1 |
| 201591_s_at | NISCH | -1.2 | -2.0 | -0.8 | -1.6 | -1.3 | -1.4 | nischarin |
| 205204_at | NMB | 2.1 | 4.9 | 1.5 | 5.5 | 2.8 | 5.6 | neuromedin B |
| 218036_x_at | NMD3 | 0.9 | 0.3 | 0.7 | 1.7 | 0.7 | 0.0 | NMD3 homolog (S. cerevisiae) |
| 203964_at | NMI | 1.8 | 1.8 | 2.1 | 2.5 | 2.3 | 2.2 | N-myc (and STAT) interactor |
| 205005_s_at | NMT2 | 1.5 | 1.5 | 1.9 | 2.6 | 0.0 | 0.0 | N-myristoyltransferase 2 |
| 205895_s_at | NOLC1 | 1.7 | 1.3 | 1.4 | 1.9 | 1.3 | 0.9 | nucleolar and coiled-body phosphoprotein 1 |
| 210756_s_at | NOTCH2 | 3.1 | 1.0 | 1.1 | 1.4 | 3.2 | 0.9 | Notch homolog 2 (Drosophila) /// Notch homolog 2 (Drosophila) |
| 213479_at | NPTX2 | 0.5 | 2.8 | 2.2 | 0.9 | 0.0 | 1.7 | neuronal pentraxin II |
| 203814_s_at | NQO2 | -2.3 | -0.6 | -1.1 | -1.8 | -1.6 | -0.7 | NAD(P)H dehydrogenase, quinone 2 |
| 207978_s_at | NR4A3 | 2.3 | 3.1 | 2.5 | 3.1 | 1.4 | 2.0 | nuclear receptor subfamily 4, group A, member 3 |
| 202395_at | NSF | 1.3 | 1.4 | 0.9 | 1.9 | 1.1 | 1.6 | N-ethylmaleimide-sensitive factor |
| 219458_s_at | NSUN3 | 1.4 | 1.6 | 1.5 | 1.8 | 1.2 | 1.2 | NOL1/NOP2/Sun domain family, member 3 |
| 203675_at | NUCB2 | -2.1 | -1.7 | -1.5 | -1.7 | -1.9 | -1.5 | nucleobindin 2 |
| 206302_s_at | NUDT4 /// NUDT4P1 | 1.5 | 1.7 | 0.9 | 1.5 | 0.9 | 0.9 | nudix (nucleoside diphosphate linked moiety X)-type motif 4 |
| 210793_s_at | NUP98 | 1.1 | 1.5 | 1.6 | 2.1 | 0.6 | 1.0 | nucleoporin 98kDa |
| 204435_at | NUPL1 | 1.6 | 1.1 | 1.9 | 1.5 | 0.8 | 0.7 | nucleoporin like 1 |
| 209629_s_at | NXT2 | 3.1 | 2.1 | 1.8 | 2.8 | 2.4 | 2.0 | nuclear transport factor 2-like export factor 2 |
| 205552_s_at | OAS1 | 2.3 | 2.0 | 3.9 | 4.7 | 2.9 | 2.3 | 2',5'-oligoadenylate synthetase 1, 40/46kDa |
| 204972_at | OAS2 | 2.6 | 2.4 | 4.0 | 4.1 | 2.8 | 2.2 | 2'-5'-oligoadenylate synthetase 2, 69/71kDa |
| 218400_at | OAS3 | 2.1 | 2.6 | 3.3 | 2.7 | 3.1 | 2.7 | 2'-5'-oligoadenylate synthetase 3, 100kDa |
| 210797_s_at | OASL | 3.4 | 3.7 | 3.8 | 4.7 | 3.9 | 3.4 | 2'-5'-oligoadenylate synthetase-like |
| 219334_s_at | OBFC2A | 2.4 | 3.8 | 2.4 | 4.1 | 1.8 | 2.4 | hypothetical protein FLJ22833 |
| 218196_at | OSTM1 | -1.4 | -1.3 | -1.2 | -0.7 | -1.1 | 0.0 | osteopetrosis associated transmembrane protein 1 |
| 207543_s_at | P4HA1 | -2.1 | -1.4 | -1.4 | -1.6 | -1.7 | -1.7 | procollagen-proline, 2-oxoglutarate 4-dioxygenase (proline 4-hydroxylase), alpha polypeptide I |
| 201651_s_at | PACSIN2 | -1.7 | -1.8 | -1.7 | -1.7 | -1.3 | -1.1 | protein kinase C and casein kinase substrate in neurons 2 |
| 200907_s_at | PALLD | 4.3 | 4.0 | 3.1 | 5.5 | 3.8 | 4.1 | palladin |
| 202336_s_at | PAM | 1.7 | 1.4 | 1.0 | 1.2 | 2.1 | 2.0 | peptidylglycine alpha-amidating monooxygenase |
| 222035_s_at | PAPOLA | 1.0 | 1.2 | 0.6 | 1.7 | 0.5 | 1.2 | poly(A) polymerase alpha |
| 218543_s_at | PARP12 | 0.4 | 0.4 | 1.3 | 1.4 | 1.0 | 1.2 | poly (ADP-ribose) polymerase family, member 12 |
| 217739_s_at | PBEF1 /// LOC646309 /// RP11-92J19.4 | 2.6 | 3.0 | 2.9 | 3.0 | 1.7 | 2.5 | pre-B-cell colony enhancing factor 1 |
| 205202_at | PCMT1 | -1.4 | -0.9 | -1.2 | -1.2 | -1.0 | 0.0 | protein-L-isoaspartate (D-aspartate) O-methyltransferase |
| 222380_s_at | PDCD6 | -0.9 | -0.7 | -0.7 | -1.7 | -1.6 | -1.0 | Similar to Microneme antigen |
| 218472_s_at | PELO | 2.2 | 3.2 | 2.3 | 2.8 | 1.4 | 2.4 | pelota homolog (Drosophila) |
| 202212_at | PES1 | 0.7 | 0.8 | 1.6 | 1.2 | 0.5 | 0.0 | pescadillo homolog 1, containing BRCT domain (zebrafish) |
| 213638_at | PHACTR1 | -1.7 | -1.5 | -1.1 | -2.4 | -2.3 | -1.7 | phosphatase and actin regulator 1 |
| 204049_s_at | PHACTR2 | 1.7 | 3.1 | 2.0 | 3.1 | 2.2 | 3.2 | phosphatase and actin regulator 2 |
| 221816_s_at | PHF11 | 1.1 | 1.2 | 2.3 | 2.4 | 1.3 | 1.4 | PHD finger protein 11 |
| 204866_at | PHF16 | 1.7 | 1.4 | 0.9 | 0.8 | 1.4 | 0.7 | PHD finger protein 16 |
| 209422_at | PHF20 | -2.2 | -2.1 | -1.6 | -1.5 | -2.0 | -1.4 | PHD finger protein 20 |
| 215236_s_at | PICALM | 4.0 | 2.7 | 2.0 | 3.3 | 3.2 | 2.1 | phosphatidylinositol binding clathrin assembly protein |
| 203879_at | PIK3CD | -2.5 | -2.1 | -2.0 | -2.0 | -1.5 | -1.4 | phosphoinositide-3-kinase, catalytic, delta polypeptide |
| 213408_s_at | PIK4CA /// LOC220686 | -1.7 | -1.4 | -1.3 | -1.7 | -1.5 | -1.2 | phosphatidylinositol 4-kinase, catalytic, alpha polypeptide /// hypothetical protein LOC220686 |
| 204269_at | PIM2 | 1.5 | 1.7 | 2.0 | 2.0 | 1.4 | 1.2 | pim-2 oncogene |
| 211205_x_at | PIP5K1A | 2.5 | 0.7 | 1.3 | 1.9 | 2.4 | 0.6 | phosphatidylinositol-4-phosphate 5-kinase, type I, alpha |
| 213111_at | PIP5K3 | 1.7 | 1.6 | 0.7 | 1.6 | 1.5 | 1.3 | phosphatidylinositol-3-phosphate/phosphatidylinositol 5-kinase, type III |
| 205273_s_at | PITRM1 | 1.9 | 1.8 | 1.4 | 2.2 | 1.9 | 1.9 | pitrilysin metallopeptidase 1 |
| 201133_s_at | PJA2 | -1.2 | -1.3 | -1.3 | -1.2 | -1.1 | -0.6 | praja 2, RING-H2 motif containing |
| 201251_at | PKM2 | 1.2 | 0.3 | 0.8 | 0.8 | 1.6 | 0.0 | pyruvate kinase, muscle |
| 210145_at | PLA2G4A | 3.4 | 4.6 | 2.9 | 3.3 | 2.3 | 3.8 | phospholipase A2, group IVA (cytosolic, calcium-dependent) |
| 219014_at | PLAC8 | -1.6 | -0.9 | -1.7 | -1.2 | -1.1 | -1.1 | placenta-specific 8 |
| 201136_at | PLP2 | -1.3 | -1.1 | -1.1 | -1.6 | -0.6 | -0.6 | proteolipid protein 2 (colonic epithelium-enriched) |
| 202446_s_at | PLSCR1 | 2.1 | 2.7 | 3.9 | 3.8 | 2.3 | 2.3 | phospholipid scramblase 1 |
| 204286_s_at | PMAIP1 | 1.6 | 2.3 | 1.3 | 2.7 | 1.3 | 1.9 | phorbol-12-myristate-13-acetate-induced protein 1 |
| 203616_at | POLB | -2.1 | -1.6 | -1.4 | -1.5 | -2.0 | -1.7 | polymerase (DNA directed), beta |
| 209382_at | POLR3C | 1.5 | 2.2 | 1.1 | 1.6 | 1.2 | 1.6 | polymerase (RNA) III (DNA directed) polypeptide C (62kD) |
| 202466_at | POLS | 1.2 | 1.4 | 1.4 | 0.9 | 0.8 | 1.1 | polymerase (DNA directed) sigma |
| 204839_at | POP5 | -1.6 | -1.2 | -1.2 | -1.0 | -1.3 | -1.1 | processing of precursor 5, ribonuclease P/MRP subunit (S. cerevisiae) |
| 220741_s_at | PPA2 | -2.3 | -1.5 | -2.0 | -1.3 | -1.9 | -1.4 | pyrophosphatase (inorganic) 2 |
| 201490_s_at | PPIF | 0.6 | 0.4 | 1.2 | 1.6 | 0.0 | 0.0 | peptidylprolyl isomerase F (cyclophilin F) |
| 37028_at | PPP1R15A | 1.0 | 1.1 | 1.3 | 1.0 | 0.9 | 0.8 | protein phosphatase 1, regulatory (inhibitor) subunit 15A |
| 202165_at | PPP1R2 | -2.3 | -0.8 | -1.1 | -1.5 | 0.0 | -0.8 | protein phosphatase 1, regulatory (inhibitor) subunit 2 |
| 203737_s_at | PPRC1 | 1.1 | 0.8 | 1.6 | 1.0 | 0.0 | 0.6 | peroxisome proliferative activated receptor, gamma, coactivator-related 1 |
| 200975_at | PPT1 | -1.6 | -1.2 | -1.3 | -1.1 | -1.5 | -0.6 | palmitoyl-protein thioesterase 1 (ceroid-lipofuscinosis, neuronal 1, infantile) |
| 201858_s_at | PRG1 | 1.0 | 1.6 | 1.1 | 2.3 | 0.9 | 1.5 | proteoglycan 1, secretory granule |
| 202742_s_at | PRKACB | -0.9 | -1.0 | -1.9 | -1.2 | -1.8 | -1.4 | protein kinase, cAMP-dependent, catalytic, beta |
| 218292_s_at | PRKAG2 | 1.5 | 1.5 | 1.1 | 1.6 | 1.6 | 1.8 | protein kinase, AMP-activated, gamma 2 non-catalytic subunit |
| 203680_at | PRKAR2B | 2.3 | 1.6 | 1.0 | 3.5 | 2.2 | 2.1 | protein kinase, cAMP-dependent, regulatory, type II, beta |
| 209685_s_at | PRKCB1 | -1.2 | -0.9 | -1.0 | -1.7 | -0.9 | -0.9 | protein kinase C, beta 1 |
| 219183_s_at | PSCD4 | -1.1 | -1.9 | -1.5 | -2.7 | 0.0 | 0.0 | pleckstrin homology, Sec7 and coiled-coil domains 4 |
| 201317_s_at | PSMA2 | 0.9 | 1.2 | 1.2 | 1.8 | 0.9 | 1.0 | proteasome (prosome, macropain) subunit, alpha type, 2 |
| 209853_s_at | PSME3 | 1.9 | 0.8 | 1.2 | 1.8 | 1.5 | 0.5 | proteasome (prosome, macropain) activator subunit 3 (PA28 gamma; Ki) |
| 212016_s_at | PTBP1 | 1.8 | 0.4 | 0.7 | 1.4 | 1.6 | 0.0 | polypyrimidine tract binding protein 1 |
| 204897_at | PTGER4 | 1.6 | 2.3 | 2.2 | 2.8 | 1.2 | 1.7 | prostaglandin E receptor 4 (subtype EP4) |
| 204748_at | PTGS2 | 3.3 | 3.7 | 4.0 | 5.0 | 0.0 | 2.5 | prostaglandin-endoperoxide synthase 2 (prostaglandin G/H synthase and cyclooxygenase) |
| 200730_s_at | PTP4A1 | 1.5 | 1.7 | 0.9 | 1.5 | 1.1 | 1.1 | protein tyrosine phosphatase type IVA, member 1 |
| 208617_s_at | PTP4A2 | -1.4 | -1.6 | -1.1 | -1.5 | -1.2 | -0.8 | protein tyrosine phosphatase type IVA, member 2 |
| 212640_at | PTPLB | -1.3 | -1.3 | -2.1 | -1.7 | -1.5 | -1.3 | protein tyrosine phosphatase-like (proline instead of catalytic arginine), member b |
| 202716_at | PTPN1 | 1.0 | 1.6 | 0.8 | 0.4 | 0.8 | 1.3 | protein tyrosine phosphatase, non-receptor type 1 |
| 212587_s_at | PTPRC | -2.0 | -1.6 | -2.8 | -1.8 | -1.4 | -0.7 | protein tyrosine phosphatase, receptor type, C |
| 221840_at | PTPRE | -3.7 | -2.8 | -2.5 | -3.9 | 0.0 | -3.0 | protein tyrosine phosphatase, receptor type, E |
| 200677_at | PTTG1IP | -2.3 | -2.3 | -2.3 | -2.2 | -1.9 | -1.8 | pituitary tumor-transforming 1 interacting protein |
| 204020_at | PURA | -1.2 | -1.6 | -1.4 | -1.0 | -1.4 | -1.4 | purine-rich element binding protein A |
| 202754_at | R3HDM1 | -1.2 | -2.2 | -1.7 | -1.6 | -0.9 | -1.0 | R3H domain containing 1 |
| 212561_at | RAB6IP1 | 2.0 | 1.9 | 2.0 | 2.0 | 1.7 | 1.7 | RAB6 interacting protein 1 |
| 218699_at | RAB7L1 | 2.2 | 2.9 | 2.8 | 2.9 | 2.1 | 2.7 | RAB7, member RAS oncogene family-like 1 |
| 219210_s_at | RAB8B | 3.1 | 3.1 | 2.3 | 3.1 | 2.5 | 2.5 | RAB8B, member RAS oncogene family |
| 221808_at | RAB9A | 2.6 | 4.0 | 3.3 | 4.1 | 2.5 | 3.8 | RAB9A, member RAS oncogene family |
| 203136_at | RABAC1 | -1.5 | -0.9 | -0.7 | -0.8 | -1.1 | -0.5 | Rab acceptor 1 (prenylated) |
| 74694_s_at | RABEP2 /// LOC652743 | -0.9 | -0.9 | -0.8 | -0.7 | 0.0 | -0.4 | rabaptin, RAB GTPase binding effector protein 2 |
| 212646_at | RAFTLIN | 0.5 | 1.5 | 0.9 | 0.9 | 0.0 | 1.1 | raft-linking protein |
| 202583_s_at | RANBP9 | 2.0 | 1.2 | 1.3 | 1.5 | 1.8 | 0.8 | RAN binding protein 9 |
| 202362_at | RAP1A | -1.9 | -1.3 | -1.0 | -1.1 | -1.4 | -1.7 | RAP1A, member of RAS oncogene family |
| 209444_at | RAP1GDS1 | -1.7 | -1.6 | -2.2 | -1.4 | -1.1 | -0.9 | RAP1, GTP-GDP dissociation stimulator 1 |
| 203185_at | RASSF2 | -2.9 | -2.7 | -2.0 | -2.6 | -2.9 | -3.0 | Ras association (RalGDS/AF-6) domain family 2 |
| 210371_s_at | RBBP4 | -1.3 | -1.4 | -1.6 | -1.4 | -1.1 | -1.4 | retinoblastoma binding protein 4 |
| 203344_s_at | RBBP8 | 1.4 | 1.6 | 1.3 | 1.3 | 0.9 | 1.0 | retinoblastoma binding protein 8 |
| 201486_at | RCN2 | -1.5 | -1.5 | -2.1 | -1.3 | -1.3 | 0.0 | reticulocalbin 2, EF-hand calcium binding domain |
| 212397_at | RDX | 1.4 | 1.6 | 1.4 | 1.6 | 1.4 | 1.4 | radixin |
| 208873_s_at | REEP5 | -2.3 | -2.0 | -1.8 | -2.2 | -1.7 | -1.4 | chromosome 5 open reading frame 18 |
| 206036_s_at | REL | 2.4 | 3.2 | 3.1 | 2.9 | 2.1 | 2.7 | v-rel reticuloendotheliosis viral oncogene homolog (avian) |
| 219041_s_at | REPIN1 | -2.9 | -3.5 | -2.6 | -2.7 | -2.2 | -2.5 | replication initiator 1 |
| 208070_s_at | REV3L | 2.3 | 2.6 | 1.5 | 2.2 | 0.8 | 2.1 | REV3-like, catalytic subunit of DNA polymerase zeta (yeast) |
| 218194_at | REXO2 | -1.6 | -1.5 | -1.6 | -2.0 | -1.1 | -0.8 | REX2, RNA exonuclease 2 homolog (S. cerevisiae) |
| 209568_s_at | RGL1 | 1.0 | 2.2 | 1.7 | 2.3 | 1.4 | 2.0 | ral guanine nucleotide dissociation stimulator-like 1 |
| 202388_at | RGS2 | -4.9 | -2.6 | -2.7 | -4.4 | -5.9 | -3.1 | regulator of G-protein signalling 2, 24kDa |
| 219045_at | RHOF | -2.0 | -2.1 | -0.5 | -0.9 | -1.1 | -1.8 | ras homolog gene family, member F (in filopodia) |
| 209545_s_at | RIPK2 | 2.7 | 2.3 | 2.2 | 2.4 | 2.3 | 1.8 | receptor-interacting serine-threonine kinase 2 |
| 209882_at | RIT1 | 1.5 | 2.2 | 1.7 | 2.3 | 1.0 | 1.7 | Ras-like without CAAX 1 |
| 218247_s_at | RKHD2 | -1.0 | -1.1 | -1.1 | -1.2 | -1.6 | -1.3 | ring finger and KH domain containing 2 |
| 201779_s_at | RNF13 | 1.9 | 1.0 | 0.9 | 1.3 | 1.3 | 0.8 | ring finger protein 13 |
| 217865_at | RNF130 | -1.5 | -1.3 | -1.8 | -1.4 | -1.4 | -1.2 | ring finger protein 130 |
| 201528_at | RPA1 | -1.1 | -2.1 | -2.8 | -0.6 | -0.7 | -1.2 | replication protein A1, 70kDa |
| 212590_at | RRAS2 | 1.9 | 0.5 | 1.7 | 2.0 | 1.7 | 0.0 | related RAS viral (r-ras) oncogene homolog 2 |
| 213797_at | RSAD2 | 3.5 | 4.5 | 4.8 | 8.0 | 4.5 | 5.2 | radical S-adenosyl methionine domain containing 2 |
| 201980_s_at | RSU1 | -2.0 | -1.4 | -1.6 | -1.3 | -1.3 | -1.1 | Ras suppressor protein 1 |
| 204198_s_at | RUNX3 | 1.1 | 0.3 | 0.6 | 0.9 | 1.8 | 0.9 | runt-related transcription factor 3 |
| 219691_at | SAMD9 | 2.2 | 2.4 | 2.7 | 3.4 | 2.6 | 2.1 | sterile alpha motif domain containing 9 |
| 220330_s_at | SAMSN1 | 2.9 | 5.6 | 4.5 | 4.9 | 2.4 | 5.3 | SAM domain, SH3 domain and nuclear localisation signals, 1 |
| 201543_s_at | SAR1A | 1.0 | 1.2 | 1.3 | 1.9 | 0.8 | 1.3 | SAR1 gene homolog A (S. cerevisiae) |
| 218254_s_at | SAR1B | 1.2 | 1.5 | 1.1 | 1.6 | 0.8 | 1.3 | SAR1 gene homolog B (S. cerevisiae) |
| 218854_at | SART2 | 2.5 | 2.3 | 1.3 | 2.0 | 2.0 | 2.0 | squamous cell carcinoma antigen recognized by T cells 2 |
| 213988_s_at | SAT | 0.8 | 2.0 | 2.0 | 1.3 | 1.0 | 1.8 | spermidine/spermine N1-acetyltransferase |
| 203408_s_at | SATB1 | 1.5 | 1.4 | 0.8 | 0.9 | 0.8 | 0.9 | special AT-rich sequence binding protein 1 (binds to nuclear matrix/scaffold-associating DNA's) |
| 211423_s_at | SC5DL | -1.8 | -1.3 | -0.3 | -0.8 | -1.4 | -0.7 | sterol-C5-desaturase (ERG3 delta-5-desaturase homolog, fungal)-like |
| 216899_s_at | SCAP2 | -1.4 | -1.3 | -1.2 | -0.8 | -1.0 | -1.3 | src family associated phosphoprotein 2 |
| 212589_at | SCP2 | 1.6 | 2.0 | 1.5 | 2.0 | 0.0 | 0.0 | Sterol carrier protein 2 |
| 212902_at | SEC24A | 3.6 | 3.0 | 2.4 | 3.4 | 3.0 | 2.7 | SEC24 related gene family, member A (S. cerevisiae) |
| 202375_at | SEC24D | 0.7 | 1.1 | 0.6 | 2.2 | 0.7 | 0.0 | SEC24 related gene family, member D (S. cerevisiae) |
| 217811_at | SELT | 1.2 | 1.2 | 1.3 | 2.2 | 1.2 | 0.9 | selenoprotein T |
| 57703_at | SENP5 | 1.2 | 0.9 | 1.5 | 1.6 | 1.0 | 1.1 | SUMO1/sentrin specific peptidase 5 |
| 208939_at | SEPHS1 | -1.6 | -1.3 | -1.5 | -1.4 | -0.9 | -1.0 | selenophosphate synthetase 1 |
| 200961_at | SEPHS2 | -1.5 | -0.9 | -1.6 | -1.0 | -1.7 | -0.7 | selenophosphate synthetase 2 |
| 212268_at | SERPINB1 | 1.6 | 1.7 | 0.7 | 1.1 | 1.7 | 2.4 | serpin peptidase inhibitor, clade B (ovalbumin), member 1 |
| 209722_s_at | SERPINB9 | 3.4 | 2.7 | 1.9 | 2.5 | 3.5 | 2.7 | serpin peptidase inhibitor, clade B (ovalbumin), member 9 |
| 202656_s_at | SERTAD2 | -0.6 | -1.2 | -1.0 | -0.9 | -0.8 | -0.9 | SERTA domain containing 2 |
| 200892_s_at | SFRS10 | 1.3 | 1.0 | 1.1 | 1.6 | 0.9 | 0.8 | splicing factor, arginine/serine-rich 10 (transformer 2 homolog, Drosophila) |
| 209339_at | SIAH2 | 2.4 | 3.0 | 1.5 | 2.5 | 2.2 | 2.5 | seven in absentia homolog 2 (Drosophila) /// seven in absentia homolog 2 (Drosophila) |
| 202255_s_at | SIPA1L1 | 0.8 | 1.6 | 3.5 | 1.8 | 0.5 | 1.6 | signal-induced proliferation-associated 1 like 1 |
| 202897_at | SIRPA | 2.2 | 2.3 | 2.4 | 3.2 | 2.5 | 2.3 | protein tyrosine phosphatase, non-receptor type substrate 1 |
| 219159_s_at | SLAMF7 | 1.7 | 2.1 | 1.4 | 2.2 | 1.7 | 1.7 | SLAM family member 7 |
| 218066_at | SLC12A7 | -2.2 | -1.4 | -0.8 | -2.3 | -3.1 | -2.1 | solute carrier family 12 (potassium/chloride transporters), member 7 |
| 212811_x_at | SLC1A4 | -1.4 | -0.4 | -1.1 | -1.5 | -0.6 | 0.0 | solute carrier family 1 (glutamate/neutral amino acid transporter), member 4 |
| 201920_at | SLC20A1 | -3.6 | -3.7 | -2.8 | -3.5 | -4.4 | -3.8 | solute carrier family 20 (phosphate transporter), member 1 |
| 217122_s_at | SLC35E2 | -0.9 | -1.6 | -1.8 | -1.7 | -0.8 | -1.3 | solute carrier family 35, member E2 |
| 218237_s_at | SLC38A1 | -0.8 | -1.4 | -1.3 | -1.9 | -0.8 | -2.2 | solute carrier family 38, member 1 |
| 210692_s_at | SLC43A3 | 2.2 | 1.5 | 2.3 | 2.1 | 2.2 | 1.1 | solute carrier family 43, member 3 |
| 205920_at | SLC6A6 | 3.0 | 1.9 | 1.6 | 5.2 | 3.2 | 1.4 | solute carrier family 6 (neurotransmitter transporter, taurine), member 6 |
| 212295_s_at | SLC7A1 | 2.0 | 1.8 | 1.4 | 1.5 | 1.8 | 1.8 | solute carrier family 7 (cationic amino acid transporter, y+ system), member 1 |
| 207528_s_at | SLC7A11 | 2.8 | 3.5 | 2.5 | 4.0 | 2.3 | 2.9 | solute carrier family 7, (cationic amino acid transporter, y+ system) member 11 |
| 201195_s_at | SLC7A5 | -0.9 | -1.4 | -1.1 | -2.1 | -0.8 | -2.1 | solute carrier family 7 (cationic amino acid transporter, y+ system), member 5 |
| 202983_at | SMARCA3 | -0.8 | -2.0 | -1.5 | -0.8 | -0.7 | -1.0 | SWI/SNF related, matrix associated, actin dependent regulator of chromatin, subfamily a, member 3 |
| 212577_at | SMCHD1 | 1.9 | 1.0 | 1.0 | 1.5 | 2.0 | 1.0 | structural maintenance of chromosomes flexible hinge domain containing 1 |
| 218327_s_at | SNAP29 | -2.0 | -1.8 | -1.8 | -1.6 | -1.3 | -0.8 | synaptosomal-associated protein, 29kDa |
| 204466_s_at | SNCA | -1.6 | -1.7 | -1.6 | -1.4 | -0.6 | 0.0 | synuclein, alpha (non A4 component of amyloid precursor) |
| 201522_x_at | SNRPN /// SNURF | -2.3 | -1.5 | -1.5 | -1.9 | -2.2 | -1.3 | small nuclear ribonucleoprotein polypeptide N /// SNRPN upstream reading frame |
| 215223_s_at | SOD2 | 1.1 | 1.1 | 2.4 | 2.5 | 0.4 | 1.0 | superoxide dismutase 2, mitochondrial |
| 202863_at | SP100 | 1.8 | 1.8 | 1.5 | 2.0 | 2.0 | 1.9 | nuclear antigen Sp100 |
| 208012_x_at | SP110 | 1.5 | 1.7 | 2.9 | 3.1 | 1.7 | 1.8 | SP110 nuclear body protein |
| 212466_at | SPRED2 | 2.3 | 1.1 | 1.8 | 1.2 | 2.6 | 0.0 | sprouty-related, EVH1 domain containing 2 |
| 203127_s_at | SPTLC2 | 1.2 | 1.2 | 0.7 | 1.3 | 1.5 | 1.6 | serine palmitoyltransferase, long chain base subunit 2 |
| 213112_s_at | SQSTM1 | 0.8 | 2.0 | 2.7 | 2.3 | 0.0 | 0.0 | sequestosome 1 |
| 203182_s_at | SRPK2 | -1.7 | -1.2 | -2.2 | -1.0 | -0.8 | -1.0 | SFRS protein kinase 2 |
| 221753_at | SSH1 | -1.6 | -1.6 | -0.9 | -1.2 | -1.6 | -1.1 | slingshot homolog 1 (Drosophila) |
| 206925_at | ST8SIA4 | 3.9 | 4.9 | 3.6 | 4.2 | 3.6 | 4.2 | ST8 alpha-N-acetyl-neuraminide alpha-2,8-sialyltransferase 4 |
| 209969_s_at | STAT1 | 1.1 | 0.9 | 3.9 | 3.0 | 1.4 | 1.1 | signal transducer and activator of transcription 1, 91kDa |
| 205170_at | STAT2 | 2.2 | 0.9 | 1.3 | 2.2 | 2.5 | 1.2 | signal transducer and activator of transcription 2, 113kDa |
| 206118_at | STAT4 | 2.9 | 3.7 | 3.1 | 3.5 | 2.2 | 3.4 | signal transducer and activator of transcription 4 |
| 203010_at | STAT5A | 3.5 | 3.6 | 2.9 | 3.9 | 3.5 | 3.0 | signal transducer and activator of transcription 5A |
| 202693_s_at | STK17A | 1.5 | 2.8 | 2.0 | 1.7 | 0.6 | 1.2 | serine/threonine kinase 17a (apoptosis-inducing) |
| 205214_at | STK17B | 1.7 | 2.0 | 1.2 | 1.9 | 0.7 | 0.0 | serine/threonine kinase 17b (apoptosis-inducing) |
| 202951_at | STK38 | -2.0 | -1.8 | -1.9 | -1.8 | -1.7 | -1.7 | serine/threonine kinase 38 |
| 211085_s_at | STK4 | 2.5 | 3.2 | 2.4 | 3.0 | 2.2 | 3.0 | serine/threonine kinase 4 /// serine/threonine kinase 4 |
| 212631_at | STX7 | -2.2 | -2.4 | -2.9 | -2.0 | -1.7 | -1.7 | Syntaxin 7 |
| 200740_s_at | SUMO3 | -2.0 | -1.7 | -1.8 | -1.9 | -1.7 | -1.6 | SMT3 suppressor of mif two 3 homolog 3 (yeast) |
| 209025_s_at | SYNCRIP | 1.1 | 1.2 | 0.9 | 1.8 | 0.7 | 1.2 | synaptotagmin binding, cytoplasmic RNA interacting protein |
| 201079_at | SYNGR2 | -3.0 | -2.5 | -1.5 | -2.6 | -2.6 | -2.3 | synaptogyrin 2 |
| 202168_at | TAF9 | 2.0 | 1.9 | 1.6 | 1.6 | 1.2 | 1.3 | TAF9 RNA polymerase II, TATA box binding protein (TBP)-associated factor, 32kDa |
| 210978_s_at | TAGLN2 | -1.7 | -1.9 | -1.6 | -2.5 | -1.4 | -1.8 | transgelin 2 |
| 210458_s_at | TANK | 1.0 | 0.6 | 0.6 | 2.7 | 1.0 | 0.4 | TRAF family member-associated NFKB activator |
| 202307_s_at | TAP1 | 1.2 | 1.1 | 1.7 | 1.5 | 1.7 | 1.5 | transporter 1, ATP-binding cassette, sub-family B (MDR/TAP) |
| 204769_s_at | TAP2 | 1.5 | 1.5 | 1.4 | 1.6 | 1.8 | 1.9 | transporter 2, ATP-binding cassette, sub-family B (MDR/TAP) |
| 209154_at | TAX1BP3 | -2.0 | -0.9 | -1.4 | -0.8 | -1.3 | -0.5 | Tax1 (human T-cell leukemia virus type I) binding protein 3 |
| 202819_s_at | TCEB3 | 1.1 | 1.5 | 1.8 | 1.9 | 0.7 | 0.9 | transcription elongation factor B (SIII), polypeptide 3 (110kDa, elongin A) |
| 216037_x_at | TCF7L2 | 3.0 | 2.8 | 3.5 | 3.7 | 1.7 | 2.5 | transcription factor 7-like 2 (T-cell specific, HMG-box) |
| 213361_at | TDRD7 | 1.6 | 1.4 | 2.1 | 2.3 | 2.3 | 1.9 | tudor domain containing 7 |
| 221035_s_at | TEX14 | 5.1 | 5.1 | 5.5 | 4.4 | 3.4 | 4.4 | testis expressed sequence 14 |
| 206715_at | TFEC | 1.2 | 2.1 | 2.0 | 2.1 | 0.9 | 1.1 | transcription factor EC |
| 209278_s_at | TFPI2 | 5.6 | 6.9 | 7.5 | 10.2 | 4.6 | 6.3 | tissue factor pathway inhibitor 2 |
| 201506_at | TGFBI | -3.2 | -1.9 | -1.9 | -3.7 | -2.6 | -1.7 | transforming growth factor, beta-induced, 68kDa |
| 203313_s_at | TGIF | 2.0 | 2.6 | 1.3 | 1.3 | 1.6 | 2.3 | TGFB-induced factor (TALE family homeobox) |
| 213191_at | TICAM1 | 1.2 | 1.7 | 1.3 | 0.9 | 0.9 | 1.7 | toll-like receptor adaptor molecule 1 |
| 212665_at | TIPARP | 1.1 | 1.8 | 0.8 | 1.4 | 0.9 | 1.7 | TCDD-inducible poly(ADP-ribose) polymerase |
| 203221_at | TLE1 | 2.9 | 1.9 | 2.0 | 1.6 | 2.4 | 1.7 | transducin-like enhancer of split 1 (E(sp1) homolog, Drosophila) |
| 202475_at | TMEM147 | -1.9 | -1.6 | -0.9 | -1.1 | -1.6 | -0.4 | seven transmembrane domain protein |
| 219690_at | **TMEM149** | -2.0 | -1.6 | -1.6 | -1.6 | -1.3 | -1.0 | U2(RNU2) small nuclear RNA auxiliary factor 1-like 4 |
| 218770_s_at | TMEM39B | -1.4 | -1.8 | -0.8 | -1.5 | -1.3 | -1.3 | transmembrane protein 39B |
| 200847_s_at | TMEM66 | -1.7 | -1.5 | -1.3 | -1.5 | -1.4 | -1.1 | transmembrane protein 66 |
| 207113_s_at | TNF | 2.5 | 2.3 | 4.2 | 4.1 | 1.3 | 1.5 | tumor necrosis factor (TNF superfamily, member 2) |
| 202643_s_at | TNFAIP3 | 1.7 | 1.9 | 2.3 | 1.8 | 1.1 | 1.2 | tumor necrosis factor, alpha-induced protein 3 |
| 206026_s_at | TNFAIP6 | 0.9 | 1.3 | 1.6 | 1.1 | 0.0 | 0.0 | tumor necrosis factor, alpha-induced protein 6 |
| 208296_x_at | TNFAIP8 | 1.5 | 2.4 | 1.6 | 1.1 | 1.5 | 2.0 | tumor necrosis factor, alpha-induced protein 8 |
| 207536_s_at | TNFRSF9 | 0.6 | 1.7 | 1.5 | 1.4 | 0.8 | 2.2 | tumor necrosis factor receptor superfamily, member 9 |
| 202687_s_at | TNFSF10 | 2.6 | 3.3 | 5.2 | 5.4 | 3.4 | 4.1 | tumor necrosis factor (ligand) superfamily, member 10 |
| 210314_x_at | TNFSF13 /// TNFSF12-TNFSF13 | -1.9 | -1.7 | -1.1 | -1.6 | -1.4 | -1.7 | tumor necrosis factor (ligand) superfamily, member 13 /// tumor necrosis factor (ligand) superfamily, member 12-member 13 |
| 206907_at | TNFSF9 | 2.2 | 2.4 | 2.2 | 2.4 | 2.4 | 1.8 | tumor necrosis factor (ligand) superfamily, member 9 |
| 209226_s_at | TNPO1 | 1.7 | 1.8 | 1.7 | 1.4 | 1.2 | 1.8 | transportin 1 |
| 208901_s_at | TOP1 | 1.4 | 2.1 | 1.6 | 1.1 | 0.5 | 1.2 | topoisomerase (DNA) I |
| 209593_s_at | TOR1B | 0.8 | 1.0 | 0.8 | 1.4 | 1.4 | 1.5 | torsin family 1, member B (torsin B) |
| 200822_x_at | TPI1 | -2.2 | -1.4 | -1.2 | -2.0 | -2.0 | -1.4 | triosephosphate isomerase 1 |
| 205599_at | TRAF1 | 2.6 | 2.3 | 2.3 | 3.3 | 2.4 | 2.3 | TNF receptor-associated factor 1 |
| 202837_at | TRAFD1 | 1.2 | 1.8 | 1.6 | 1.4 | 1.0 | 1.1 | TRAF-type zinc finger domain containing 1 |
| 204341_at | TRIM16 /// TRIM16L /// LOC653524 | 1.0 | 1.2 | 1.3 | 1.8 | 1.0 | 1.2 | tripartite motif-containing 16 /// similar to tripartite motif-containing 16; estrogen-responsive B box protein |
| 204804_at | TRIM21 | 1.9 | 1.6 | 1.6 | 2.4 | 2.1 | 1.8 | tripartite motif-containing 21 |
| 213293_s_at | TRIM22 | 1.3 | 0.9 | 1.3 | 1.6 | 1.3 | 0.9 | tripartite motif-containing 22 |
| 218617_at | TRIT1 | -1.4 | -1.7 | -1.1 | -1.1 | -1.2 | -1.4 | tRNA isopentenyltransferase 1 |
| 221987_s_at | TSR1 | 1.4 | 1.3 | 1.1 | 2.0 | 0.9 | 0.0 | hypothetical protein FLJ10534 |
| 217964_at | TTC19 | -1.7 | -1.3 | -1.6 | -1.5 | -1.1 | -0.9 | tetratricopeptide repeat domain 19 |
| 208073_x_at | TTC3 | -1.3 | -1.5 | -1.4 | -1.8 | -1.1 | -1.1 | tetratricopeptide repeat domain 3 |
| 218442_at | TTC4 | 1.7 | 1.5 | 2.0 | 1.6 | 1.1 | 1.2 | tetratricopeptide repeat domain 4 |
| 46167_at | TTC4 /// C1orf175 | 1.1 | 1.3 | 1.7 | 1.4 | 0.5 | 1.2 | tetratricopeptide repeat domain 4 /// chromosome 1 open reading frame 175 |
| 212320_at | TUBB | -1.0 | -0.8 | -1.5 | -1.0 | -1.0 | 0.0 | tubulin, beta polypeptide |
| 214729_at | TWISTNB | 4.4 | 2.2 | 2.0 | 1.8 | 0.0 | 0.0 | TWIST neighbor |
| 201581_at | TXNDC13 | -1.1 | -1.3 | -1.6 | -1.7 | 0.0 | -0.7 | thioredoxin domain containing 13 |
| 221253_s_at | TXNDC5 | -0.8 | -1.5 | -1.5 | -1.6 | -0.6 | -1.1 | thioredoxin domain containing 5 /// thioredoxin domain containing 5 |
| 201266_at | TXNRD1 | 1.6 | 1.8 | 1.3 | 1.7 | 1.3 | 1.5 | thioredoxin reductase 1 |
| 201898_s_at | UBE2A | 1.2 | 1.4 | 1.0 | 1.4 | 1.2 | 1.2 | ubiquitin-conjugating enzyme E2A (RAD6 homolog) |
| 217826_s_at | UBE2J1 | -3.2 | -2.8 | -3.8 | -3.1 | -2.4 | -2.9 | ubiquitin-conjugating enzyme E2, J1 (UBC6 homolog, yeast) |
| 201649_at | UBE2L6 | 0.8 | 1.0 | 2.1 | 1.9 | 1.6 | 1.3 | ubiquitin-conjugating enzyme E2L 6 |
| 201534_s_at | UBL3 | 1.3 | 1.6 | 0.8 | 1.2 | 1.1 | 1.6 | ubiquitin-like 3 |
| 208998_at | UCP2 | -2.9 | -2.6 | -3.0 | -3.5 | -2.2 | -2.0 | uncoupling protein 2 (mitochondrial, proton carrier) |
| 204881_s_at | UGCG | -2.0 | -1.5 | -0.9 | -1.5 | -1.9 | -1.8 | UDP-glucose ceramide glucosyltransferase |
| 213327_s_at | USP12 | 1.7 | 1.1 | 1.7 | 1.6 | 1.4 | 0.9 | ubiquitin specific peptidase 12 |
| 201672_s_at | USP14 | 1.1 | 1.6 | 1.3 | 1.4 | 1.0 | 1.6 | ubiquitin specific peptidase 14 (tRNA-guanine transglycosylase) |
| 219211_at | USP18 | 3.0 | 3.6 | 6.1 | 7.5 | 4.0 | 4.1 | ubiquitin specific peptidase 18 |
| 220419_s_at | USP25 | 1.2 | 1.3 | 1.0 | 1.2 | 1.7 | 1.7 | ubiquitin specific peptidase 25 |
| 202546_at | VAMP8 | -3.8 | -3.0 | -3.5 | -2.9 | -3.0 | -2.3 | vesicle-associated membrane protein 8 (endobrevin) |
| 208623_s_at | VIL2 | -1.8 | -0.5 | -0.6 | -0.9 | -1.5 | 0.0 | villin 2 (ezrin) |
| 213243_at | VPS13B | -1.2 | -1.0 | -0.9 | -2.4 | -1.4 | -1.3 | vacuolar protein sorting 13B (yeast) |
| 217821_s_at | WBP11 | 1.5 | 0.7 | 0.9 | 0.7 | 1.2 | 0.0 | WW domain binding protein 11 |
| 214662_at | WDR43 | 1.6 | 1.2 | 1.6 | 0.9 | 0.9 | 1.0 | WD repeat domain 43 |
| 221712_s_at | WDR74 | 1.1 | 1.8 | 1.5 | 1.7 | 0.0 | 1.4 | WD repeat domain 74 /// WD repeat domain 74 |
| 209045_at | XPNPEP1 | -1.5 | -1.5 | -1.2 | -0.9 | -0.7 | 0.0 | X-prolyl aminopeptidase (aminopeptidase P) 1, soluble |
| 215207_x_at | YDD19 /// C6orf68 /// LOC389850 /// LOC654174 | 1.3 | 1.5 | 1.2 | 1.6 | 0.0 | 0.0 | YDD19 protein /// chromosome 6 open reading frame 68 /// similar to hypothetical protein, MGC:7199 |
| 210317_s_at | YWHAE | 2.5 | 0.6 | 0.7 | 1.3 | 2.4 | 0.0 | tyrosine 3-monooxygenase/tryptophan 5-monooxygenase activation protein, epsilon polypeptide |
| 217741_s_at | ZA20D2 | 1.6 | 1.4 | 1.0 | 1.1 | 1.4 | 0.7 | zinc finger, A20 domain containing 2 |
| 219312_s_at | ZBTB10 | 2.5 | 2.6 | 1.5 | 1.5 | 1.4 | 1.4 | zinc finger and BTB domain containing 10 |
| 204182_s_at | ZBTB43 | 1.1 | 1.5 | 1.5 | 2.0 | 1.1 | 1.1 | zinc finger protein 297B |
| 220104_at | ZC3HAV1 | 1.5 | 2.5 | 2.0 | 2.2 | 1.3 | 2.8 | zinc finger CCCH-type, antiviral 1 |
| 219062_s_at | ZCCHC2 | 1.4 | 1.6 | 1.4 | 1.8 | 1.6 | 1.8 | zinc finger, CCHC domain containing 2 |
| 211962_s_at | ZFP36L1 | 2.6 | 3.4 | 1.4 | 2.5 | 2.4 | 2.7 | zinc finger protein 36, C3H type-like 1 |
| 219778_at | ZFPM2 | 8.5 | 4.4 | 2.6 | 7.9 | 7.2 | 4.0 | zinc finger protein, multitype 2 |
| 201856_s_at | ZFR | 1.8 | 0.5 | 0.7 | 0.6 | 1.7 | 0.6 | zinc finger RNA binding protein |
| 203556_at | ZHX2 | 1.5 | 1.9 | 1.1 | 1.0 | 0.6 | 1.3 | zinc fingers and homeoboxes 2 |
| 200828_s_at | ZNF207 | 1.9 | 1.0 | 1.0 | 1.9 | 1.4 | 1.0 | zinc finger protein 207 |
| 212774_at | ZNF238 | -3.0 | -3.2 | -2.5 | -3.6 | -2.7 | -3.3 | zinc finger protein 238 |
| 203248_at | ZNF24 | 1.5 | 0.7 | 0.8 | 1.3 | 1.6 | 0.7 | zinc finger protein 24 (KOX 17) |
| 219540_at | ZNF267 | 0.4 | 1.4 | 1.5 | 1.6 | 0.5 | 1.1 | zinc finger protein 267 |
| 212368_at | ZNF292 | -1.3 | -1.8 | -1.8 | -1.6 | -1.2 | -2.0 | zinc finger protein 292 |
| 212742_at | ZNF364 | 1.5 | 1.7 | 1.4 | 2.1 | 1.5 | 1.5 | zinc finger protein 364 |
| 205739_x_at | ZNF588 | 0.8 | 0.4 | 1.5 | 1.8 | 1.0 | 1.0 | zinc finger protein 588 |
| 211950_at | ZUBR1 | 1.4 | 1.1 | 1.3 | 1.1 | 1.4 | 1.1 | retinoblastoma-associated factor 600 |

Values are Log2 fold change in expression. Fold change in expression was determined with respect to vehicle treated samples from the same donor. D1, D2, D3, and D4 represent samples from different blood donors. Genes were selected if the Affymetrix probes sets had signal detection p-value less than 0.01 and an expression change p-value less than 0.005 across all replicates for at least one of the TLR agonists. Dashed lines in the Gene Symbol and Gene Title columns represent Affymetrix probes with no gene Symbol or known annotation.
