# Supplementary figures and images for: Transcriptional networks in plasmacytoid dendritic cells stimulated with synthetic TLR 7 agonists
Source: BMC Immunol. 2007 Oct 12;8:26. doi: 10.1186/1471-2172-8-26 (PMC2175514; doi:10.1186/1471-2172-8-26)

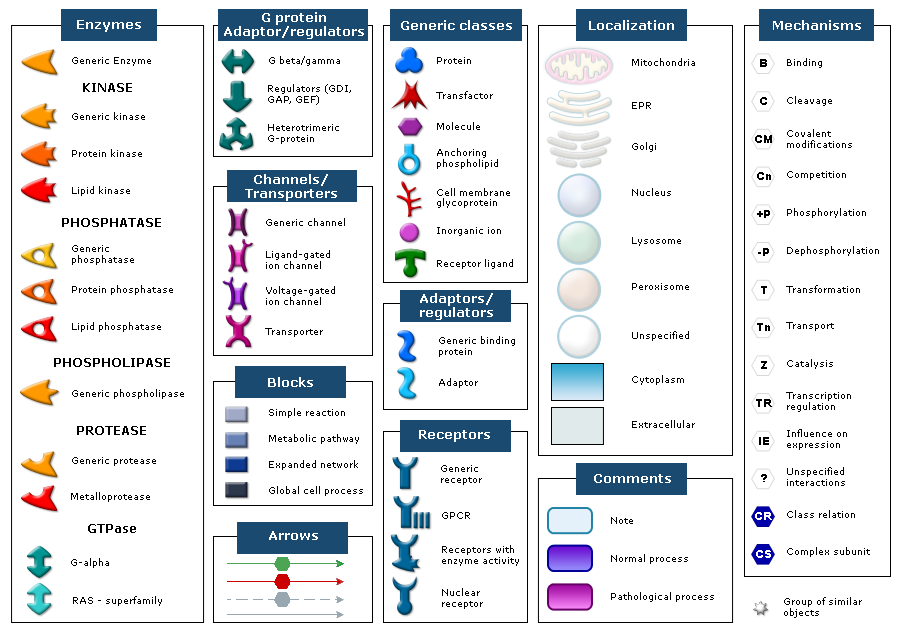

Supplement: Additional file 2 — Legend describing symbols used in networks. Legend provides a key to the symbols used in the MetaCore networks. [file 1471-2172-8-26-S2.png]
